# Type I IFN–dependent Fc**γ**RIV signaling in murine monocytes promotes lethal anaphylaxis during viral infections

**DOI:** 10.1172/JCI192371

**Published:** 2026-02-16

**Authors:** Abdelrahman Elwy, Hossam Abdelrahman, Julia Specht, Gina M. Ewert, Justa Friebus-Kardash, Swati Dhiman, Julia Falkenstein, Theresa Charlotte Christ, Elisa Wiebeck, Arzoo Shamoon, Nils B. Leimkühler, Thomas Gramberg, Alina Russ, Ulrich Kalinke, Fei Kuang, Kathrin Sutter, Manfred Kopf, Matthias Mack, Wiebke Hansen, Falk Nimmerjahn, Karl S. Lang

**Affiliations:** 1 Institute of Immunology, University Hospital Essen, Medical Faculty, University of Duisburg-Essen, Essen, Germany.; 2 Department of Nephrology, University Hospital Essen, and; 3 Institute of Medical Microbiology, University Hospital Essen, University of Duisburg-Essen, Essen, Germany.; 4 Department of Hematology and Stem Cell Transplantation, University Hospital Essen, Essen, Germany.; 5 Harald zur Hausen Institute of Virology, Friedrich-Alexander-Universität Erlangen-Nürnberg, Erlangen, Germany.; 6 Institute for Experimental Infection Research, TWINCORE, Centre for Experimental and Clinical Infection Research, a joint venture between the Helmholtz Centre for Infection Research and the Hannover Medical School, Hannover, Germany.; 7 Institute for Virology and; 8 Institute for The Research on HIV and AIDS-associated Diseases, University Hospital Essen, University of Duisburg-Essen, Essen, Germany.; 9 Institute of Molecular Health Sciences, Department of Biology, ETH Zürich, Zürich, Switzerland.; 10 Department of Nephrology, University Hospital Regensburg, Regensburg, Germany.; 11 Division of Genetics, Department of Biology, University of Erlangen-Nürnberg, Erlangen, Germany.

**Keywords:** Autoimmunity, Immunology, Infectious disease, Allergy, COVID-19, Innate immunity

## Abstract

Anaphylaxis is a life-threatening hypersensitivity reaction. Clinical observations suggest heightened susceptibility during viral infections, yet the mechanisms remain poorly defined. Here, we show that both active and passive IgG-mediated anaphylaxis were exacerbated in the setting of acute viral infection. In mice, this enhancement was driven predominantly by FcγRIV, the homolog of human FcγRIIIa. FcγRIV crosslinking induced anaphylactic symptoms selectively in infected animals, with no effect in naive conditions. Among leukocytes, inflammatory monocytes emerged as the principal drivers of this lethal reaction. Viral infection triggered a strong upregulation of FcγRIV on inflammatory monocytes, an effect absent in type I IFN receptor–deficient (*Ifnar1*-deficient) mice. Extending these findings, we observed increased frequencies of CD16-expressing classical monocytes in patients with acute COVID-19, and murine SARS-CoV-2 infection recapitulated this phenotype. Mechanistically, FcγRIV crosslinking during infection promoted the production of platelet-activating factor, the key mediator of mortality, in a type I IFN–dependent (IFN-I–dependent) manner. Together, these findings indicate that viral infection creates an immune milieu that heightens monocyte sensitivity to Fcγ receptor engagement, positioning these cells as major effectors of IgG-mediated hypersensitivity in the infected host. They further suggest that Fc receptor pathway modulation merits further investigation in contexts with heightened IFN-I responses, such as in systemic lupus erythematosus.

## Abstract 2

FcγRIV-mediated anaphylaxis during viral infection is driven by IFN-I-dependent FcγRIV upregulation on inflammatory monocytes.

## Graphical abstract

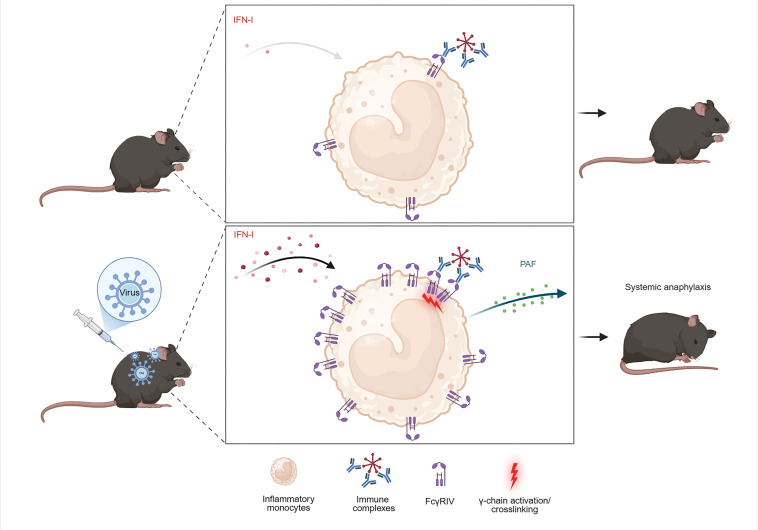

## Introduction

Anaphylaxis is a severe, potentially life-threatening hypersensitivity reaction characterized by rapid onset and systemic involvement. While it is commonly associated with allergens such as foods, insect venoms, or drugs, emerging evidence suggests that viral infections may increase the susceptibility to anaphylaxis (1–5). This heightened risk during infections has been noted clinically (4), yet the mechanisms by which viral infection modifies anaphylactic responsiveness remain poorly understood.

Classical anaphylaxis is predominantly mediated by IgE antibodies, which bind to high-affinity FcεRI on mast cells and basophils, leading to degranulation and the release of histamine and other mediators (6). In contrast, nonclassical or alternative pathways involve IgG antibodies that interact with low-affinity Fcγ receptors (FcγRs), particularly human FcγRIIIa (CD16a) expressed on monocytes, neutrophils, macrophages, as well as mast cells (7). This pathway can also induce systemic anaphylaxis through the production of platelet-activating factor (PAF), largely bypassing mast cell–dependent mechanisms (8). While classical IgE-mediated anaphylaxis is well characterized, the nonclassical pathway’s contribution, particularly during immune dysregulation such as viral infections, remains underexplored.

Recent studies have highlighted the role of FcγRs in mediating immune responses, including hypersensitivity reactions (9). Among these, the human FcγRIIIa (encoded by *CD16A*) and its murine homolog FcγRIV (encoded by *Fcgr4*) are implicated in the modulation of allergic and inflammatory pathways (10–12). It is important to note that FcγR nomenclature differs between species, with human FcγRIIIa being functionally homologous to murine FcγRIV (9). In humans, FcγRIIIa has been linked to IgG-mediated autoimmune reactions (7, 13, 14), while FcγRIV, in particular, has been shown to play a critical role in antibody-dependent cellular responses during inflammation (11). However, whether FcγRIV contributes to IgG-mediated hypersensitivity in vivo and how this may be influenced by infection-associated immune changes, have not been directly investigated.

Monocytes are key innate immune cells that play a central role in FcγR-mediated signaling and immune regulation. During viral infections, inflammatory monocytes are rapidly recruited to sites of infection, where they contribute to both protective and pathological immune responses. In diseases such as dengue, influenza, and COVID-19, these cells have been implicated in driving excessive inflammation (15, 16). Owing to their prominent role in FcγR signaling and effector functions (17), monocytes are well positioned to influence hypersensitivity reactions under infection-associated immune priming.

IFN-Is, critical antiviral cytokines, profoundly shape immune responses by modulating the activation and function of various immune cells, including monocytes (18, 19). Despite this, the effect of IFN-I on FcγR regulation remains poorly understood. Elevated IFN-I levels have been associated with autoimmune diseases such as systemic lupus erythematosus, in which FcγR-mediated pathways contribute to disease pathology (20). The interplay between IFN-I signaling, FcγR expression, and immune effector functions during viral infections, particularly in shaping hypersensitivity reactions, remains an area of active investigation.

Emerging evidence suggests that viral infections can modulate immune responsiveness in ways that may enhance susceptibility to IgG-mediated hypersensitivity reactions (21, 22). However, the mechanisms underlying this increased risk remain elusive. In this study, we investigate the mechanisms underlying the heightened anaphylactic response observed during viral infections. We identify FcγRIV as a critical mediator of this response and demonstrate its selective involvement in infected, but not naive, conditions. Our findings revealed that monocytes, rather than granulocytes or macrophages, were the primary effectors of FcγRIV-driven anaphylaxis, with PAF emerging as a central mediator. Furthermore, we show that the upregulation of FcγRIV on inflammatory monocytes was IFN-I dependent, linking viral infection–induced immune modulation to hypersensitivity pathology. These insights advance our understanding of FcγRs-mediated immune responses and have what we believe to be important implications for therapeutic strategies targeting FcγRs in autoimmune and infectious diseases.

## Results

### Passive systemic anaphylaxis demonstrates FcγRIV-dependent exacerbation in infected mice.

**Figure 1:**
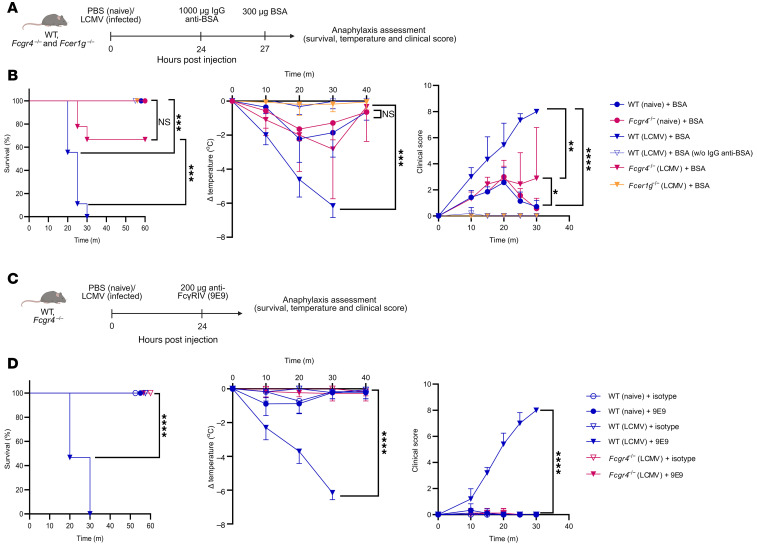
Passive systemic anaphylaxis demonstrates FcγRIV-dependent exacerbation in infected mice. (**A**) Schematic of the passive systemic anaphylaxis (PSA) model used in **B**. (**B**) Naive WT and *Fcgr4^–/–^* mice, as well as LCMV-infected WT, *Fcgr4^–/–^*, and *Fcer1g^–/–^* mice (1 × 10^6^ PFU LCMV) were treated 24 hours post infection (h.p.i.) with 1,000 μg anti-BSA IgG followed 3 hours later by 300 μg BSA. One infected WT group received BSA alone. Survival, body temperature, and clinical score (see Supplemental Table 1) were monitored over time. Data were pooled from 2 independent experiments (*n* = 3–5 mice/group/experiment), except the BSA-only group (*n* = 5; 1 experiment). (**C**) Schematic of the PSA model used in **D**. (**D**) Naive and LCMV-infected WT or *Fcgr4^–/–^* mice (1 × 10^6^ PFU LCMV) were treated i.v. 24 h.p.i. with the FcγRIV-specific antibody 9E9 or an isotype control (200 μg). Survival, body temperature, and clinical score were monitored over time. Results show pooled data from 3 independent experiments with similar results (*n* = 3–5 mice/group/experiment). All data are presented as the mean ± SD. *P < 0.05, **P < 0.01, ***P < 0.001, and ****P < 0.0001, by log-rank test for survival (**B** and **D**) and 2-way ANOVA for clinical score and body temperature (**B** and **D**).

To investigate the effect of viral infections on susceptibility to anaphylaxis, we utilized a mouse model of the acute lymphocytic choriomeningitis virus (LCMV-WE, hereafter referred to as LCMV) infection. Passive systemic anaphylaxis was initially induced by injecting BSA into mice that had been treated with anti-BSA IgG antibodies (Figure 1A) as previously reported (23). Interestingly, infected mice developed severe anaphylactic symptoms, including profound hypothermia, worse clinical scores, and increased mortality, whereas naive mice exhibited only mild hypothermia (Figure 1B). The effect observed in infected mice was predominantly mediated by FcγRIV, as FcγRIV-deficient (*Fcgr4^–/–^*) mice showed markedly attenuated responses. In line with previous reports (24, 25), mice lacking all activating IgG and IgE receptors (*FcRγ^–/–^*, officially named *Fcer1g^–/–^* mice) were completely resistant, displaying no anaphylactic symptoms. Moreover, LCMV-infected mice treated with BSA without receiving anti-BSA IgG showed no symptoms.

To directly evaluate the involvement of FcγRIV in driving these responses, we used an FcγRIV-specific antibody (9E9). Consistent with the BSA model, infected mice treated with the 9E9 antibody developed severe anaphylactic symptoms, including hypothermia, and increased mortality, while naive mice were unaffected (Figure 1, C and D). Next, to ensure that the observed effects were primarily dependent on FcγRIV crosslinking, we considered the possibility that the Fc portion of the crosslinking antibody might engage FcγRIII, as previously reported (26). To address this, we performed additional experiments using FcγRIII-deficient mice (*Fcgr3^–/–^*). These mice exhibited anaphylactic symptoms comparable to those of WT control mice, with only a minor delay of a few minutes in response onset, confirming that FcγRIV activation was the dominant driver under infection-associated immune priming (Supplemental Figure 1A; supplemental material available online with this article; https://doi.org/10.1172/JCI192371DS1). These findings indicate that the heightened susceptibility to passive IgG-mediated anaphylaxis during viral infection was mediated via FcγRIV.

To assess whether the severity of anaphylactic responses depended on the dose of the crosslinking antibody, we injected mice with either 20 μg or 200 μg 9E9 antibody. Both doses led to comparable mortality rates, although symptoms appeared a few minutes later in the lower-dose treatment group, suggesting that within this range, FcγRIV engagement threshold rather than dose magnitude governs outcomes (Supplemental Figure 1B).

To further validate the model and ensure the robustness of our observations, we compared the effects of i.v. and i.p. administration of the 9E9 antibody. Both routes of administration elicited similar mortality rates and anaphylactic symptoms in infected mice, demonstrating the reproducibility of the model (Supplemental Figure 1C). Interestingly, the onset of symptoms following i.p. injection occurred approximately 20 minutes later than with i.v. injection, likely due to the slower systemic absorption associated with the i.p. route. This comparison highlights the versatility of the model and confirms that the observed effects were independent of the injection route.

### FcγRIV activation upon infection drives robust systemic inflammatory response.

**Figure 2:**
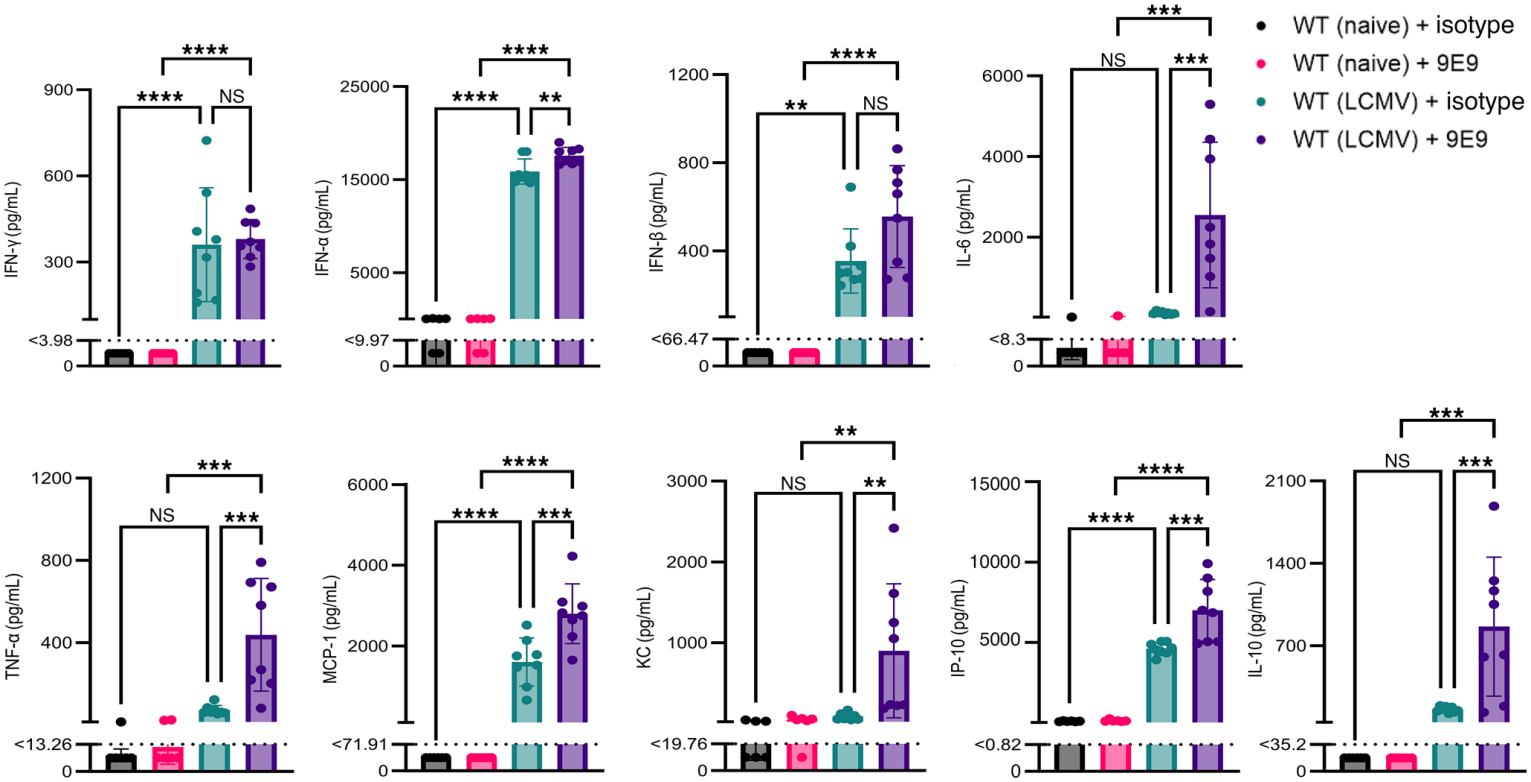
FcγRIV activation upon infection drives a robust systemic inflammatory response. WT naive and LCMV-infected mice (1 × 10^6^ PFU) were treated i.p. 24 h.p.i. with the FcγRIV-specific antibody 9E9 (200 μg) or with an isotype control antibody. Serum was collected 30 minutes after treatment, and the levels of IFN-γ, IFN-α, IFN-β, IL-10, IL-6, TNF-α, MCP-1, monocyte chemoattractant protein-1; KC, keratinocytes-derived chemokine, and IP-10 were measured using LEGENDplex. Dotted lines indicate the lower limit of detection (LOD) for each analyte. Values below the LOD were assigned a value of half the detection limit for visualization and statistical analysis. Data were pooled from 2 independent experiments (*n* = 3–4 mice/group/experiment). Data are presented as the mean ± SD. **P < 0.01, ***P < 0.001, and ****P < 0.0001, by 1-way ANOVA.

To investigate the cytokine milieu associated with FcγRIV crosslinking, we measured the levels of multiple cytokines and chemokines in the serum of naive and infected WT mice treated with the crosslinking antibody (9E9) or isotype control. Notably, infected mice exhibited a marked increase in proinflammatory cytokines, including IFN-γ, IFN-α, IFN-β, IL-6, TNF-α, MCP-1, KC, and IP-10, following FcγRIV crosslinking compared with isotype-treated controls (Figure 2). Among these, IL-6, TNF-α, and MCP-1 levels were particularly elevated, suggesting robust activation of an inflammatory cascade. Interestingly, IL-10 levels also increased in infected mice after FcγRIV crosslinking, potentially reflecting a compensatory regulatory mechanism. In contrast, cytokine induction in naive mice treated with the 9E9 antibody was negligible and comparable to that in isotype controls across all measured cytokines. This dichotomy reinforces the infection-specific nature of the FcγRIV-mediated response. Collectively, these findings indicate that FcγRIV crosslinking in an infection-primed immune environment triggered a pronounced inflammatory cytokine profile.

### IFN-I is required for FcγRIV-dependent anaphylaxis after infection.

**Figure 3:**
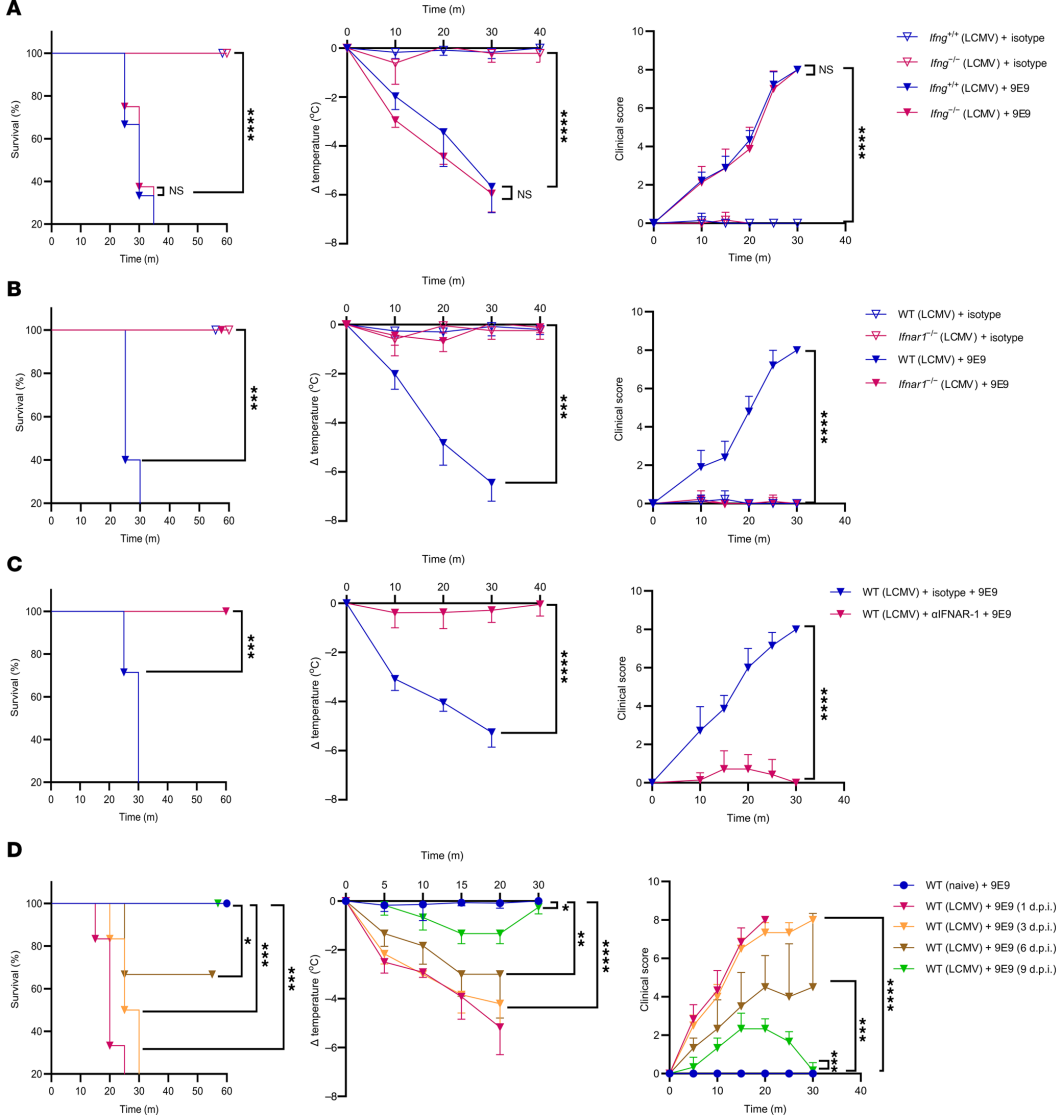
IFN-I is required for FcγRIV-dependent anaphylaxis after infection. (**A**) LCMV-infected *Ifng^+/+^* and *Ifng^–/–^* mice (1 × 10^6^ PFU) were treated 24 h.p.i. with 9E9 (FcγRIV-specific antibody, 200 μg) or an isotype control. Survival, body temperature and clinical score (Supplemental Table 1) were monitored over time. Data were pooled from 2 independent experiments (*n* = 3–5 mice/group/experiment). (**B**) LCMV-infected WT and *Ifnar1^–/–^* mice (1 × 10^6^ PFU) were treated 24 h.p.i. with 9E9 or an isotype control (200 μg). Survival, body temperature, and clinical score were monitored over time. Data were pooled from 3 independent experiments (*n* = 3–5 mice/group/experiment). (**C**) WT mice were pretreated 5 hours before LCMV infection (1 × 10^6^ PFU) with MAR1-5A3 (IFNAR1-blocking antibody, 1 mg) or mouse IgG1 isotype control, and then treated 24 h.p.i. with 9E9 (200 μg). Survival, body temperature, and clinical score were monitored over time. Data were pooled from 2 independent experiments (*n* = 3–4 mice/group/experiment). (**D**) Naive and LCMV-infected WT mice (1 × 10^6^ PFU) were treated with 9E9 (200 μg) on different days after infection (day 1, 3, 6, or 9). Survival, body temperature, and clinical score were monitored over time. Data are from 1 experiment (*n* = 6 mice/group). All data are presented as the mean ± SD. *P < 0.05, **P < 0.01, ***P < 0.001, and ****P < 0.0001, by log-rank test for survival (**A**–**D**) or 2-way ANOVA for body temperature and clinical score (**A**–**D**).

To explore the role of specific cytokines in driving FcγRIV-dependent anaphylaxis during infection, we first evaluated the contribution of IFN-γ, given its known role in regulating immune responses and modulating the expression of FcγRs, including FcγRIV (27). Since IFN-γ has been implicated in the upregulation of FcγRIV expression on immune cells, we hypothesized that it might be critical for mediating anaphylaxis upon viral infection. Using *Ifng^–/–^* mice, we observed that these animals still developed anaphylactic symptoms upon FcγRIV crosslinking, similar to WT mice, suggesting that IFN-γ was not required under these conditions (Figure 3A). In line with our earlier findings, naive *Ifng^–/–^* mice showed no symptoms upon FcγRIV crosslinking (Supplemental Figure 2A). This finding prompted us to investigate the role of IFN-I signaling, which plays a central role in antiviral immunity.

Next, we used mice lacking the IFN-I receptor (*Ifnar1^–/–^*), which are unable to respond to IFN-I signaling. Remarkably, infected *Ifnar1^–/–^* mice were completely protected from anaphylaxis, showing no mortality or other symptoms following FcγRIV crosslinking (Figure 3B). Consistently, triple-KO mice (*MyD88^–/–^* *Trif^–/–^* *Cardif^–/–^*) lacking IFN-I production (Supplemental Figure 2B) but still expressing IFNAR were also resistant to FcγRIV-mediated anaphylaxis (Supplemental Figure 2C). To further validate these findings, we transiently blocked IFN-I signaling in WT mice using an anti-IFNAR1 monoclonal antibody. Similar to the genetic ablation of *Ifnar1*, antibody-mediated IFNAR1 blockade efficiently prevented FcγRIV-induced anaphylaxis (Figure 3C), confirming that IFN-I signaling was necessary for FcγRIV-mediated responses in vivo.

To further assess the dependency on IFN-I, we examined the effect of varying levels of IFN-I production on the susceptibility to anaphylaxis. We infected mice with either a low dose or a high dose of LCMV, which induced differential IFN-I responses (Supplemental Figure 2D). Interestingly, mice infected with a low dose of LCMV did not develop anaphylactic symptoms following FcγRIV crosslinking (Supplemental Figure 2, E and F), whereas those infected with a high dose showed severe symptoms and mortality similar to our previous observations. This dose-dependent effect reinforces the idea that IFN-I levels influence the degree of immune priming and, subsequently, the susceptibility to FcγRIV-mediated anaphylaxis.

To further define the temporal window of susceptibility to FcγRIV-mediated anaphylaxis during infection, we injected naive mice or LCMV-infected mice at different time points (days 1, 3, 6, and 9 after infection) with anti-9E9 antibody. Naive mice showed no signs of anaphylaxis. In contrast, mice treated on post-infection day 1 or day 3 succumbed uniformly, displaying rapid and severe anaphylaxis. On day 6 after infection, 40% of the animals died, while survivors showed pronounced hypothermia and elevated clinical scores. By post-infection day 9, no mortality was observed, although mice still displayed mild temperature loss and subtle behavioral abnormalities (Figure 3D). To link these findings with IFN-I dynamics, we measured serum IFN-α levels over the course of infection. IFN-α peaked during the early days of infection and gradually declined by day 9 (Supplemental Figure 2G), mirroring the observed susceptibility window to FcγRIV-mediated anaphylaxis. These results indicate that susceptibility to FcγRIV-mediated anaphylaxis tracked with the infection-driven IFN-I response and did not occur once antiviral signaling waned.

**Figure 4:**
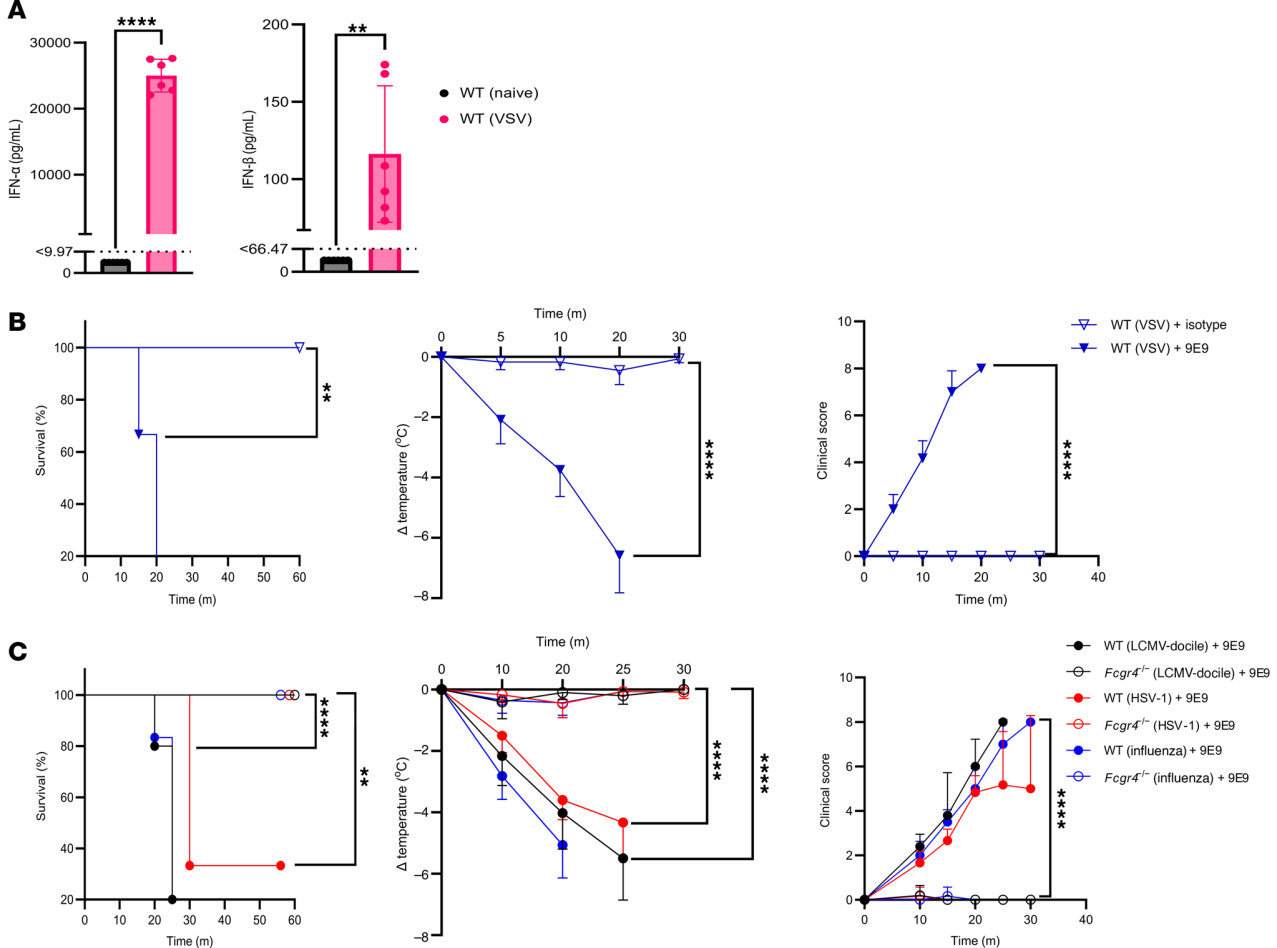
FcγRIV activation induces systemic anaphylaxis in VSV-, HSV-, and influenza A–infected mice. (**A**) Serum levels of IFN-α and IFN-β in naive and VSV-infected WT mice (1 × 10^8^ PFU). Dotted lines indicate the LOD for each analyte. Values below the LOD were assigned a value of half the detection limit for visualization and statistical analysis. Results show pooled data from 2 independent experiments with similar results (*n* = 3 mice/group/experiment). (**B**) VSV-infected WT mice (1 × 10^8^ PFU) were treated 24 h.p.i. with 9E9 or an isotype control (200 μg). Survival, body temperature, and clinical score were monitored over time. Results are representative of 2 independent experiments (*n* = 3 mice/group/experiment). (**C**) WT and *Fcgr4*^−/−^ mice were infected i.v. with LCMV-docile (5 × 10^5^ PFU), HSV-1 (1 × 10^6^ PFU), or influenza A virus (1 × 10^6^ PFU) and then treated 24 h.p.i. with 200 μg 9E9 antibody. Survival, body temperature, and clinical score were monitored over time. Results show data from 1 experiment (*n* = 5–6 mice/group). All data are presented as the mean ± SD. **P < 0.01 and ****P < 0.0001, by Student’s *t* test (**A**), log-rank test for survival (**B** and **C**), or 2-way ANOVA for body temperature and clinical score (**B** and **C**).

To extend these findings to other viral infections, we infected mice with vesicular stomatitis virus (VSV), a model in which IFN-I is highly upregulated (Figure 4A) within hours following infection (28). Similar to LCMV infection, mice infected with VSV and subsequently treated with the 9E9 antibody exhibited severe anaphylactic symptoms and mortality (Figure 4B). Similarly, mice infected with the persistent LCMV strain (LCMV-docile), herpes virus type 1 (HSV-1), or influenza A virus died upon FcγRIV crosslinking (Figure 4C). These data support the idea that IFN-I–associated immune priming, rather than virus-specific features, underlies susceptibility across models.

### IFN-I signaling is crucial for FcγRIV upregulation on inflammatory monocytes.

**Figure 5:**
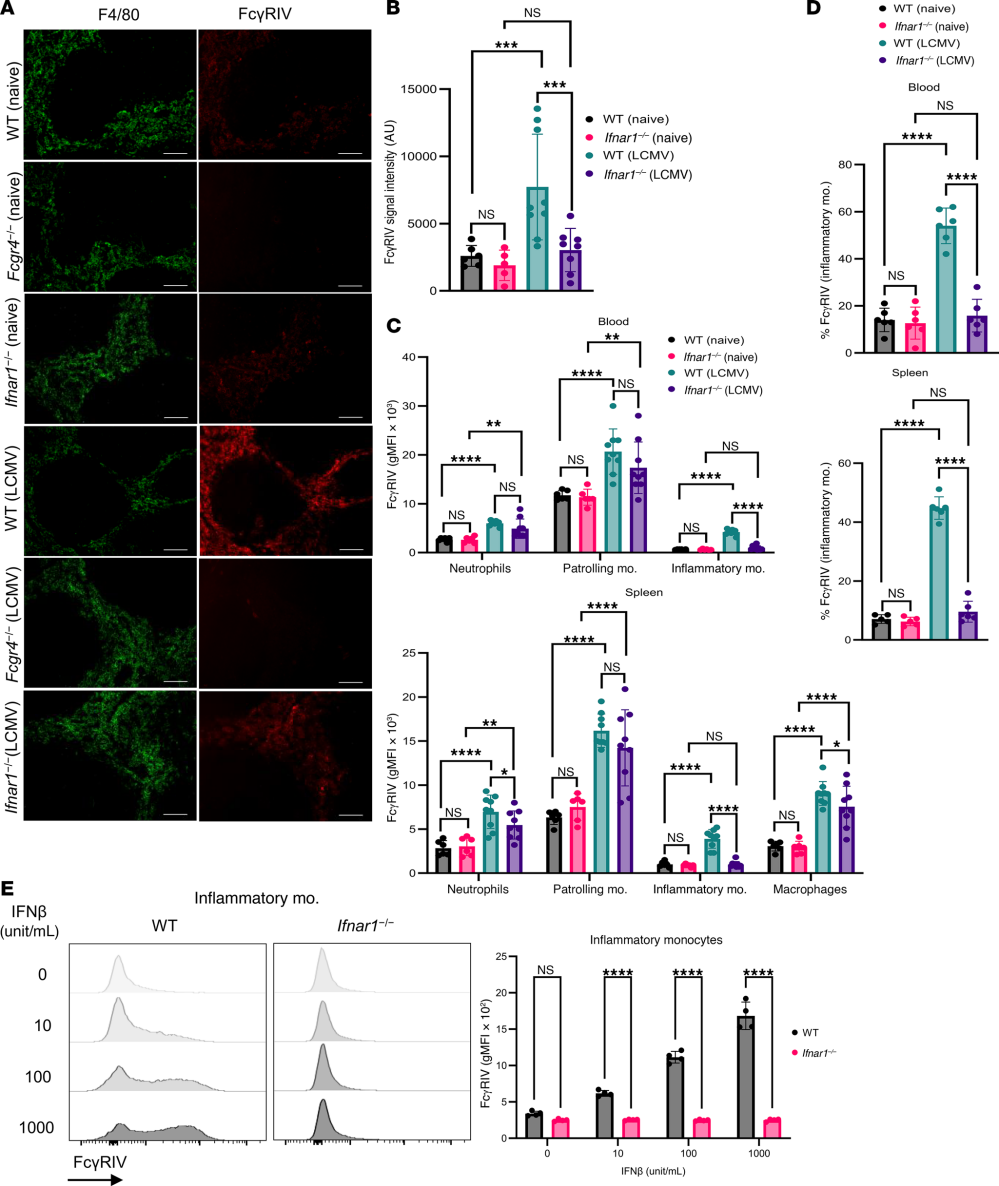
IFN-I signaling is crucial for FcγRIV upregulation on inflammatory monocytes. (**A**) IHC images of splenic tissue from naive and LCMV-infected WT, *Fcgr4^–/–^*, and *Ifnar1^–/–^* mice 24 h.p.i. Representative images from 3 independent experiments (*n* = 2–3 mice/group/experiment). Scale bars: 100 μm. (**B**) MFI quantification of FcγRIV in spleen sections of naive and LCMV-infected WT and *Ifnar1^–/–^* mice (*n* = 2–3 mice/group/experiment; data were pooled from 2–3 experiments). (**C**) Geometric mean fluorescence intensity (gMFI) of the FcγRIV expression on neutrophils (CD11b^+^Ly6G^+^), patrolling monocytes (mo.) (CD11b^+^Ly6C^–^CD43^+^FcγRIV^+^), and inflammatory monocytes (CD11b^+^Ly6C^hi^). Monocyte subsets were gated from negative lineages (Ly6G^–^CD3^–^CD19^–^) in blood, as were macrophages in spleens from naive and LCMV-infected WT and *Ifnar1^–/–^* mice (1 × 10^6^ PFU) (24 h.p.i.). Data were pooled from 2 (naive) or 3 (infected) experiments (*n* = 3 mice/group/experiment). (**D**) Quantification of FcγRIV^+^Ly6C^hi^ inflammatory monocytes in the blood and spleen of naive and LCMV-infected (1 × 10^6^ PFU) WT and *Ifnar1^–/–^* mice 24 h.p.i. Data were pooled from 2 independent experiments (*n* = 3 mice/group/experiment). (**E**) Bone marrow inflammatory monocytes from WT and *Ifnar1^–/–^* mice were treated for 18 hours with varying IFN-β concentrations. Histograms and gMFI quantification are shown. Data are shown from 2 of 3 independent experiments; all experiments produced similar results (*n* = 2 mice/group/experiment). Data are presented as the mean ± SD. *P < 0.05, **P < 0.01, ***P < 0.001, and ****P < 0.0001, by 2-way ANOVA.

To determine whether IFN-I influences FcγRIV expression during viral infection, we first assessed FcγRIV expression in splenic tissue by histology. LCMV infection markedly increased FcγRIV expression, with significant differences observed between WT and *Ifnar1^–/–^* mice (Figure 5, A and B). Next, we quantified FcγRIV expression on immune cell subsets (neutrophils, patrolling monocytes, inflammatory monocytes, and macrophages) in both blood and spleen using flow cytometry. In WT mice, viral infection induced a robust upregulation of FcγRIV specifically on inflammatory monocytes, whereas *Ifnar1^–/–^* mice showed no infection-induced FcγRIV upregulation (Figure 5C and Supplemental Figure 3A). Moreover, *Ifnar1^–/–^* mice exhibited a reduced frequency of FcγRIV-expressing inflammatory monocytes compared with WT controls (Figure 5D), reinforcing the critical role of IFN-I signaling in driving the expression of FcγRIV on inflammatory monocytes.

To confirm that this regulation is directly dependent on IFN-I signaling rather than developmental alterations in *Ifnar1^–/–^* mice, we transiently blocked IFNAR1 signaling in WT mice using a neutralizing anti-IFNAR1 antibody during LCMV infection. Antibody-mediated IFNAR1 blockade efficiently prevented the infection-induced upregulation of FcγRIV on inflammatory monocytes (Supplemental Figure 3B), fully recapitulating the phenotype observed in *Ifnar1^–/–^* mice.

Supporting this conclusion, triple-KO mice lacking IFN-I production (*MyD88^–/–^* *Trif^–/–^* *Cardif^–/–^*) also failed to upregulate FcγRIV on inflammatory monocytes (Supplemental Figure 3C). Furthermore, kinetics analysis revealed that FcγRIV expression on inflammatory monocytes closely mirrored IFN levels during the course of infection (Supplemental Figure 3D and Supplemental Figure 2G). Finally, mast cells in the blood likewise displayed IFN-I–dependent FcγRIV upregulation following infection (Supplemental Figure 3E), highlighting that IFN-I broadly regulated FcγRIV expression across multiple immune cell populations.

To directly evaluate the role of IFN-I signaling in regulating FcγRIV expression, we treated bone marrow–derived monocytes ex vivo with recombinant IFN-β. In line with our in vivo findings, FcγRIV expression was significantly upregulated on monocytes, while no notable changes were observed on *Ifnar1^–/–^* mouse–derived monocytes (Figure 5E). These results indicate that IFN-β selectively drove FcγRIV expression on monocytes, further supporting our in vivo data that IFN-I signaling was essential for the upregulation of FcγRIV on inflammatory monocytes during infection.

Together, these data highlight that IFN-I signaling is crucial for the infection-induced upregulation of FcγRIV on inflammatory monocytes. This upregulation likely plays a key role in driving the anaphylactic response observed following FcγRIV activation during viral infections.

### Inflammatory monocytes are essential mediators of anaphylaxis upon viral infection.

To identify the effector cells responsible for anaphylaxis during viral infection, we performed a series of antibody-mediated depletion and genetic KO experiments. Because mast cells are classical mediators of anaphylaxis, we first examined their role using *Kit^W-sh^-*deficient mice (Sash-KO), which lack mast cells (29). Sash mice developed anaphylaxis comparable to that seen in WT controls (Supplemental Figure 4A), excluding mast cells as critical mediators in this setting. Neutrophils have also been implicated in anaphylactic reactions (23). However, neutrophil-depleted mice also displayed responses similar to those seen in WT animals (Supplemental Figure 4, B and C), indicating that neutrophils were dispensable for the observed FcγRIV-dependent anaphylaxis.

Given the established role of macrophages in nonclassical hypersensitivity reactions (6, 30), we next assessed FcγRIV expression across splenic macrophage subsets, including marginal metallophilic macrophages (MMMs), marginal zone macrophages (MZMs), red pulp macrophages (RPMs), as well as patrolling monocytes. RPMs exhibited the highest FcγRIV expression among macrophages, which was further enhanced during infection (Supplemental Figure 5, A and B). However, depletion of phagocytic cells (monocytes/macrophages) with clodronate liposomes completely prevented anaphylaxis (Supplemental Figure 5, C and D), whereas *Spi-C*–deficient mice, which lack RPMs (31) (Supplemental Figure 6, A and B) remained fully susceptible (Supplemental Figure 6C). These data ruled out RPMs as the primary effector cells.

**Figure 6:**
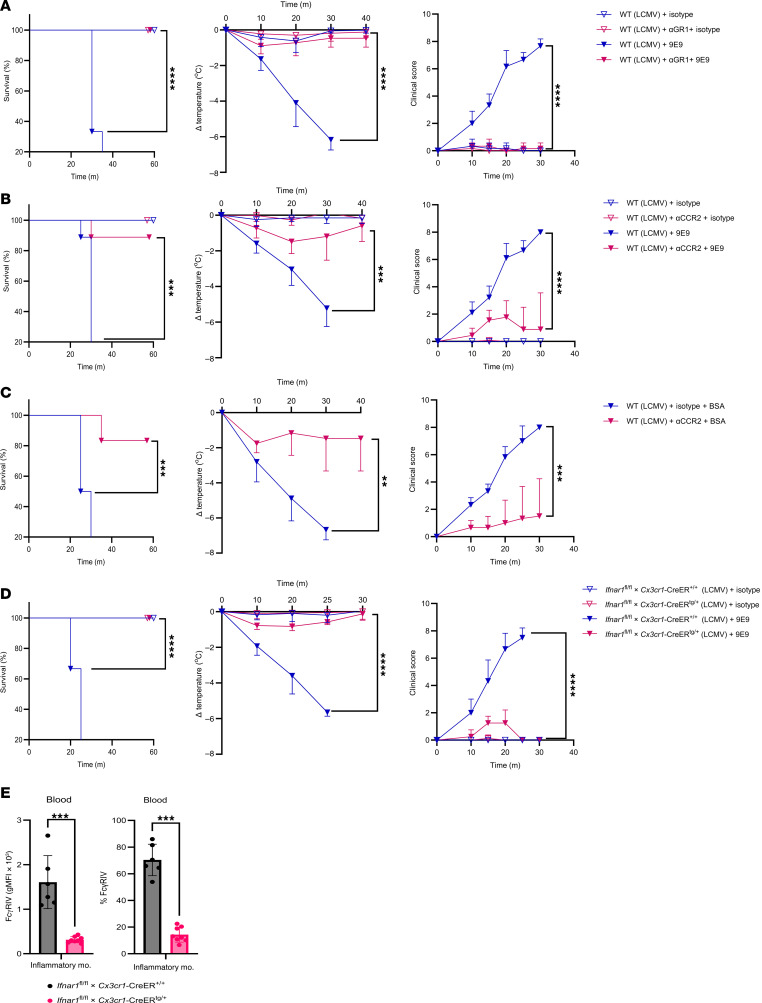
Inflammatory monocytes are essential mediators of anaphylaxis upon viral infection. (**A**) WT mice were infected with LCMV (1 × 10^6^ PFU) and treated with 9E9 (20 μg) or isotype 24 h.p.i. One group received RB6-8C5 (200 μg) 2 h.p.i. Survival, body temperature, and clinical score were monitored over time. Data were pooled from 2 experiments (*n* = 3 mice/group/experiment). (**B**) WT mice infected with LCMV (1 × 10^6^ PFU) were treated 24 h.p.i. with 9E9 (20 μg) or an isotype control. CCR2^+^ inflammatory monocytes were depleted with MC-21 antibody (35 μg) 2 h.p.i. Survival, body temperature, and clinical score were monitored over time. Data were pooled from 3 experiments (*n* = 2–3 mice/group/experiment). (**C**) LCMV-infected WT mice received PSA (anti-BSA IgG 1,000 μg + BSA 300 μg) 24 h.p.i., Some mice received MC-21 antibody. Survival, body temperature, and clinical score were monitored over time. Data were pooled from 2 experiments (*n* = 3 mice/group/experiment). (**D**) *Ifnar1^fl/fl^* *Cx3cr1*-CreER^tg/+^ mice and littermate controls were treated with tamoxifen to induce Cre recombination, infected with LCMV (1 × 10^6^ PFU), and then treated 24 h.p.i. with 9E9 (20 μg) or an isotype control. Survival, body temperature, and clinical score were monitored over time. Data represent 1 experiment (*n* = 3–4 mice/group). (**E**) gMFI and frequencies of FcγRIV on inflammatory monocytes from the blood of mice in **D**, 1 hour before antibody or isotype control treatment. Data represent 1 experiment (*n* = 6–8 mice/group). All data are presented as the mean ± SD. **P < 0.01, ***P < 0.001, and ****P < 0.0001, by log-rank test (survival) (**A**–**D**), 2-way ANOVA (clinical score and temperature) (**A**–**D**), or Student’s *t* test (**E**).

To further narrow down the responsible population, we induced depletion of GR1^+^ cells in mice, encompassing both neutrophils and inflammatory monocytes (Supplemental Figure 7A). GR1 depletion fully protected mice from anaphylaxis (Figure 6A). Since neutrophils had already been excluded (Supplemental Figure 4C), these results strongly implicated inflammatory monocytes.

Direct depletion of inflammatory monocytes using a CCR2 antibody (Supplemental Figure 7B) significantly attenuated anaphylaxis, with treated mice exhibiting only mild hypothermia and stable clinical scores following FcγRIV crosslinking during infection (Figure 6B). Comparable protection was observed in a BSA-induced anaphylaxis model (Figure 6C), demonstrating that inflammatory monocytes contributed broadly to FcγRIV-driven hypersensitivity. Supporting this conclusion, *Ifnar1^–/–^* mice — unable to upregulate FcγRIV on inflammatory monocytes — were similarly protected from anaphylaxis.

To test whether IFN-I signaling in monocytes and macrophages is required for FcγRIV-mediated anaphylaxis, we utilized *Ifnar1^fl/fl^ Cx3cr1-CreERT2* mice. Tamoxifen-induced deletion of *Ifnar1* in myeloid compartment conferred complete protection from FcγRIV-mediated anaphylaxis, with no mortality or clinical symptoms (Figure 6D). Consistently, infection-induced FcγRIV upregulation on inflammatory monocytes was abrogated in these mice (Figure 6E), confirming that IFN-I signaling in monocytes/macrophages was essential for FcγRIV expression and anaphylaxis development.

Consistent with these results, *CCR2^–/–^* mice showed partial protection, with approximately 50% mortality (Supplemental Figure 7C). As expected, these mice accumulated FcγRIV^+^ inflammatory monocytes in the bone marrow but still retained detectable circulating monocytes after infection (Supplemental Figure 7D). Thus, unlike antibody-mediated depletion, CCR2 deficiency reduced but did not eliminate inflammatory monocytes, explaining the incomplete protection. Together, these results identify inflammatory monocytes as the primary required population under infection-induced immune priming, while acknowledging that CCR2-based approaches affect additional CCR2^+^ myeloid subsets.

### SARS-CoV-2 infection drives conserved upregulation of activatory FcγRs on inflammatory monocytes.

**Figure 7:**
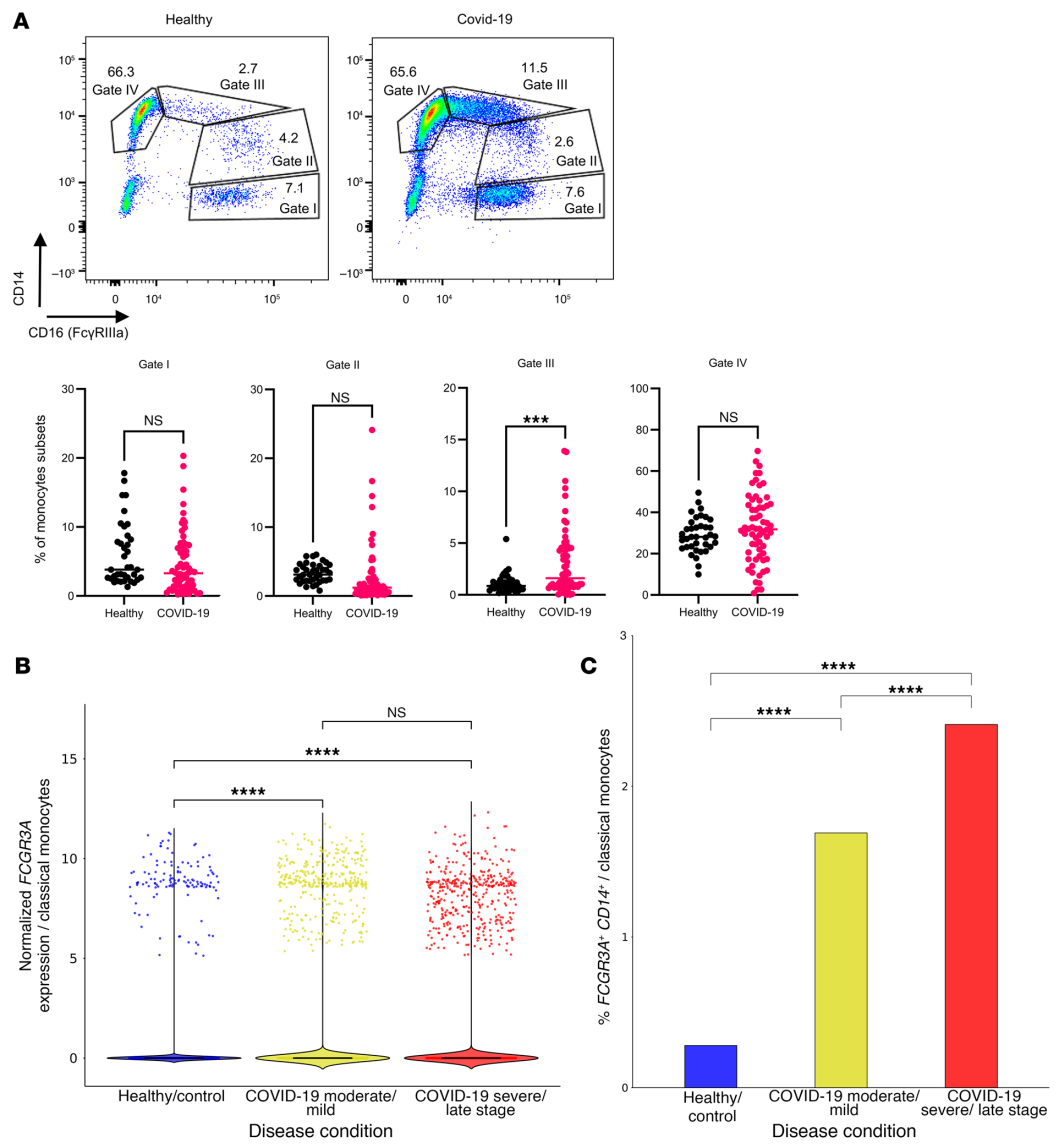
SARS-CoV-2 infection drives conserved upregulation of activatory FcγRs on inflammatory monocytes. (**A**) Representative dot plots and corresponding percentages showing CD14 and CD16 expression in gated monocytes, categorized into 4 populations (gated from CD11b^+^CD8^–^CD56^–^, not shown) isolated from peripheral blood of healthy controls (*n* = 37) and patients with COVID-19 (*n* = 64). (**B**) Normalized expression of *FCGR3A* in cells annotated as classical monocytes comparing healthy controls (mean = 0.042, SD = 0.610), patients with mild-to-moderate COVID-19 (mean = 0.214, SD = 1.364), and patients with severe COVID-19 (mean = 0.219, SD = 1.340). The dataset was obtained from publicly available scRNA-Seq data on classical monocytes (32) and analyzed. (**C**) Frequency of *CD14*^+^*FCGR3A*^+^ classical monocytes in healthy controls, patients with mild-to-moderate COVID-19, and patients with severe COVID-19. The dataset was obtained from publicly available scRNA-Seq of classical monocytes (32) and analyzed. All data are presented as the mean ± SD. ***P < 0.001 and ****P < 0.0001, by Student’s *t* test (**A**), Kruskal-Wallis test with post hoc pairwise Wilcoxon rank-sum tests (**B**), or χ^2^ test with post hoc pairwise Fisher’s exact test (**C**).

Given our findings that inflammatory monocytes act as essential mediators of anaphylaxis through FcγRIV during viral infection in mice, we sought to determine whether this phenomenon might have clinical relevance in humans. To explore this, we analyzed the monocyte subsets in peripheral blood samples from participants with acute SARS-CoV-2 infection and compared them with healthy controls. Our analysis revealed a significantly higher percentage of CD16-expressing inflammatory monocytes in SARS-CoV-2–infected patients compared with healthy individuals (Figure 7A). Moving forward, we reanalyzed publicly available single-cell RNA-Seq (scRNA-Seq) datasets of peripheral blood monocytes from patients with SARS-CoV-2 across disease severities (32). This analysis revealed a significant upregulation of *FCGR3A* expression in classical monocytes from patients with COVID-19 compared with healthy controls, with similar levels observed in both mild-to-moderate and severe cases (Figure 7B). Consistent with these transcriptional changes, the frequency of *CD14^+^FCGR3A^+^* classical monocytes was also elevated in patients with COVID-19 showing higher levels with increasing disease severity (Figure 7C).

To further examine whether SARS-CoV-2 infection drives similar changes in FcγR expression in vivo, we used the murine K18-hACE2 infection model. Flow cytometric analysis demonstrated that SARS-CoV-2 infection significantly increased both the frequency and surface expression of FcγRIV on inflammatory monocytes in the peripheral blood and spleen 48 hours after infection (Supplemental Figure 8, A and B). To determine whether this upregulation is a general feature of viral infection, we analyzed FcγRIV expression on inflammatory monocytes following HSV-1 and influenza A virus infection (Supplemental Figure 8C). Both viruses similarly induced FcγRIV expression, consistent with the susceptibility of these mice to FcγRIV-mediated anaphylaxis (Figure 4C). These findings indicate that infection-associated immune activation, rather than specific viral tropism, drives FcγR upregulation.

Together, these data show that both patients and mice with SARS-CoV-2 infection displayed infection-driven upregulation of activating FcγRs on inflammatory monocytes, mirroring the FcγRIV-dependent phenotype observed in our LCMV model. Consistent findings in HSV-1 and influenza A virus–infected mice further support a conserved mechanism whereby diverse viral infections enhance FcγR expression on monocytes, potentially predisposing to antibody-mediated immunopathology, including anaphylaxis.

### FcγRIV post-infection crosslinking triggers PAF production, leading to mortality in infected mice.

**Figure 8:**
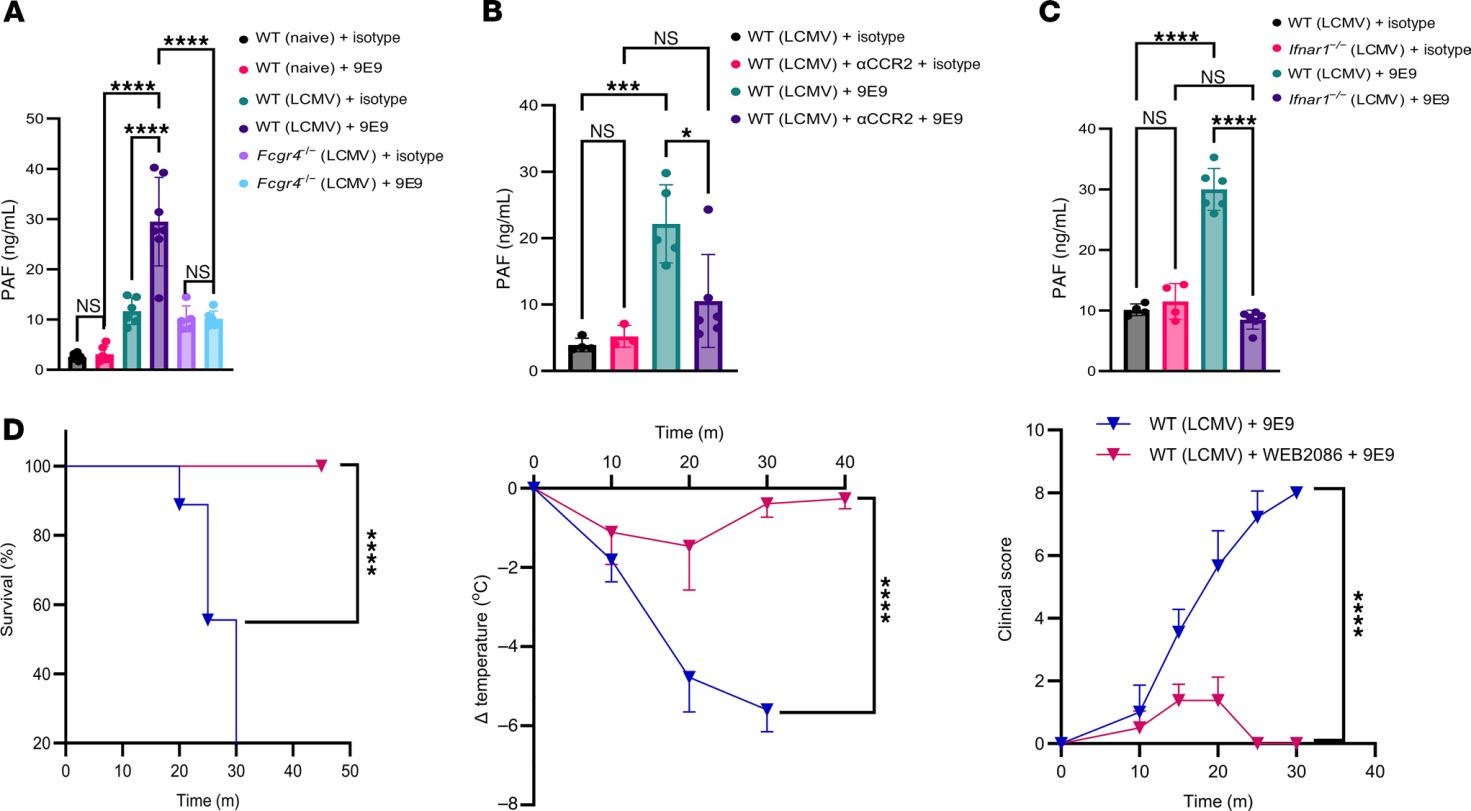
FcγRIV post-infection crosslinking triggers PAF production, leading to mortality in infected mice. (**A**) Serum PAF levels in naive WT mice and LCMV-infected WT and *Fcgr4^–/–^* mice (1 × 10^6^ PFU) 20 minutes after i.p. 9E9 (200 μg) or isotype control treatment, 24 h.p.i. Data were pooled from 2 experiments (*n =* 2–4 mice/group/experiment). (**B**) Serum PAF levels in LCMV-infected mice (1 × 10^6^ PFU) 20 minutes after i.p. 9E9 (200 μg) or isotype control treatment. Some mice received MC-21 (CCR2-depleting antibody, 35 μg) or an isotype control antibody. Data were pooled from 2 independent experiments (or 1 experiment for isotype-treated animals) with similar results (*n* = 2–3 mice/group/experiment). (**C**) Serum PAF levels in LCMV-infected (1 × 10^6^ PFU) WT and *Ifnar1^–/–^* mice, which were treated with either 9E9 (200 μg) or an isotype control 24 h.p.i. Data were pooled from 2 independent experiments with similar results (*n* = 2–3 mice/group/experiment). (**D**) Kaplan-Meier survival curves, body temperature, and clinical score (Supplemental Table 1) were used to compare LCMV-infected (1 × 10^6^ PFU) WT mice treated with 200 μg 9E9 antibody 24 h.p.i. One group received an additional treatment with a PAF-R blocker (WEB2086) (100 μg) 20 minutes before the 9E9 treatment. Data were pooled from 2 experiments (*n* = 3–4 mice/group/experiment). All data are presented as the mean ± SD. *P < 0.05, ***P < 0.001, and ****P < 0.0001, by 1-way ANOVA (**A**– **C**) or log-rank test for survival or 2-way ANOVA for body temperature and clinical score (**D**).

Given the role of PAF in mediating nonclassical anaphylaxis via FcγRs, we sought to determine PAF levels following FcγRIV crosslinking. Remarkably, FcγRIV crosslinking led to a significant increase in serum PAF levels in LCMV-infected mice but not in naive mice or infected *Fcgr4^–/–^* mice (Figure 8A). Furthermore, to confirm that PAF production was independent of FcγRIII, we measured serum PAF levels in *Fcgr3^–/–^* mice following FcγRIV crosslinking. Similar to WT mice, *Fcgr3^–/–^* mice exhibited increased PAF levels (Supplemental Figure 9A), indicating that FcγRIII did not contribute to PAF production during FcγRIV crosslinking in infected mice. These data suggest that FcγRIV leads to PAF production upon crosslinking during viral infection.

In line with our earlier findings, PAF production was significantly inhibited in both clodronate-treated mice (Supplemental Figure 9B), which lack monocytes/macrophages, and CCR2-depleted mice (Figure 8B), which lack inflammatory monocytes, as well as in *Ifnar1^–/–^* mice (Figure 8C). These results highlight the importance of both inflammatory monocytes and IFN-I signaling in driving PAF production during FcγRIV crosslinking. Consistent with this, mast cell–deficient mice, which nevertheless succumbed to FcγRIV-mediated anaphylaxis, exhibited increased PAF levels following receptor crosslinking, confirming that the observed effect was independent of mast cells (Supplemental Figure 9C).

To assess whether PAF alone mediates the anaphylactic response, we treated infected mice with WEB2086, a PAF receptor (PAF-R) blocker, prior to FcγRIV crosslinking. Notably, mice receiving the PAF-R blocker showed mild hypothermia, had normal survival rates, and exhibited no severe clinical signs of anaphylaxis compared with untreated control mice (Figure 8D). These data support the idea that PAF production is downstream of FcγRIV engagement in an infection-primed immune environment.

### Prophylactic MonoFab administration prevents IgG-mediated anaphylaxis in the infection-induced inflammatory environment.

**Figure 9:**
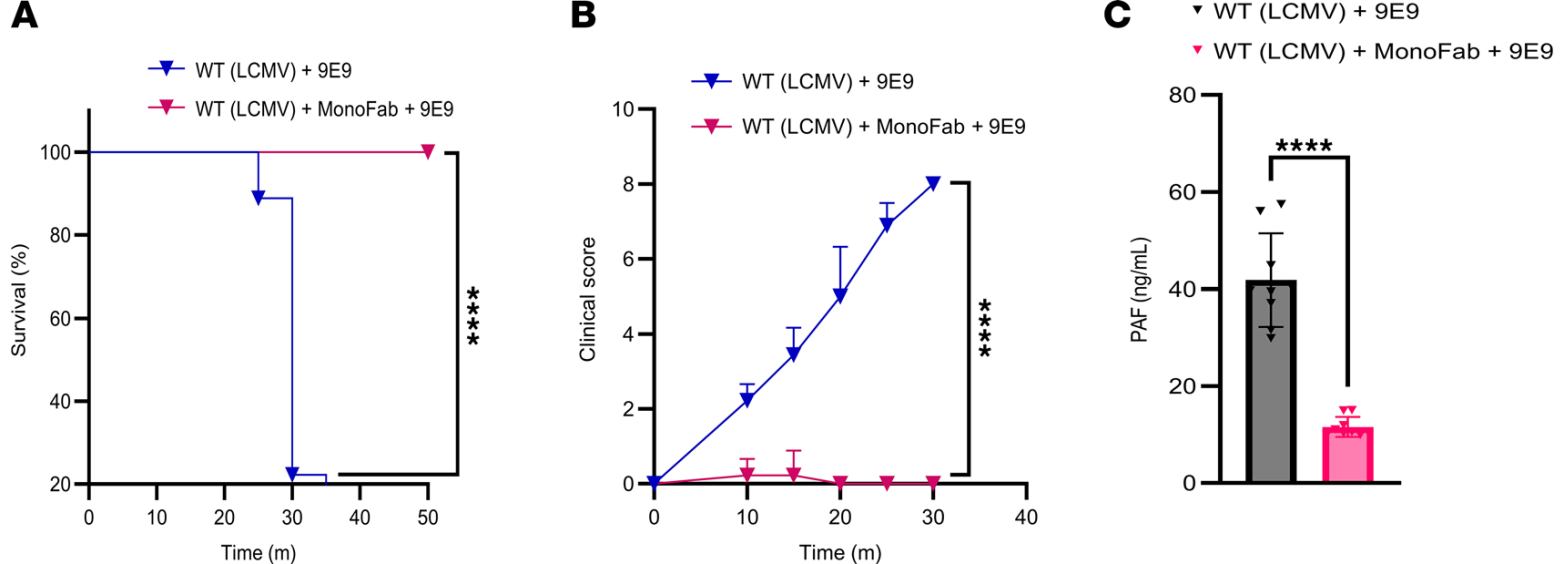
Prophylactic MonoFab administration prevents IgG-mediated anaphylaxis in the infection-induced inflammatory environment. (**A**) Kaplan-Meier survival curves were used to compare LCMV-infected (1 × 10^6^ PFU) WT mice. Mice were treated i.v. with the FcγRIV-specific antibody 9E9 (200 μg) 24 h.p.i. One group received an additional treatment with 9E9-MonoFab 20 minutes prior to the 9E9-full antibody treatment. Data were pooled from 3 independent experiments with similar results (*n* = 3 mice/group/experiment). (**B**) The clinical condition of the mice in **A** was assessed using a standardized scoring system (detailed in Supplemental Table 1). (**C**) PAF serum levels were measured in the mice in **A**. All data are presented as the mean ± SD. ****P < 0.0001, by log-rank test for survival (**A**), 2-way ANOVA (**B**), or Student’s *t* test (**C**).

Given the acute nature of anaphylaxis and the clinical importance of developing Fc blockers for treating IgG-mediated autoimmune diseases, we explored whether blocking FcγRIV with MonoFab, (the monovalent Fab fragment of the anti-FcγRIV antibody 9E9) prior to FcγRIV crosslinking and subsequent anaphylactic events could deliver beneficial outcomes. Indeed, treating infected mice with MonoFab against FcγRIV effectively inhibited the anaphylactic response. MonoFab-treated mice showed significantly improved clinical scores and enhanced survival compared with untreated controls (Figure 9, A and B). Consistent with these findings, MonoFab treatment also resulted in a marked reduction in serum PAF levels (Figure 9C), aligning with our previous observations that PAF is a critical mediator of anaphylaxis in this model. These results highlight the therapeutic potential of MonoFab in mitigating FcγRIV-driven anaphylaxis during viral infections.

### Viral infection shifts FcγR dependency toward FcγRIV in active systemic anaphylaxis.

**Figure 10:**
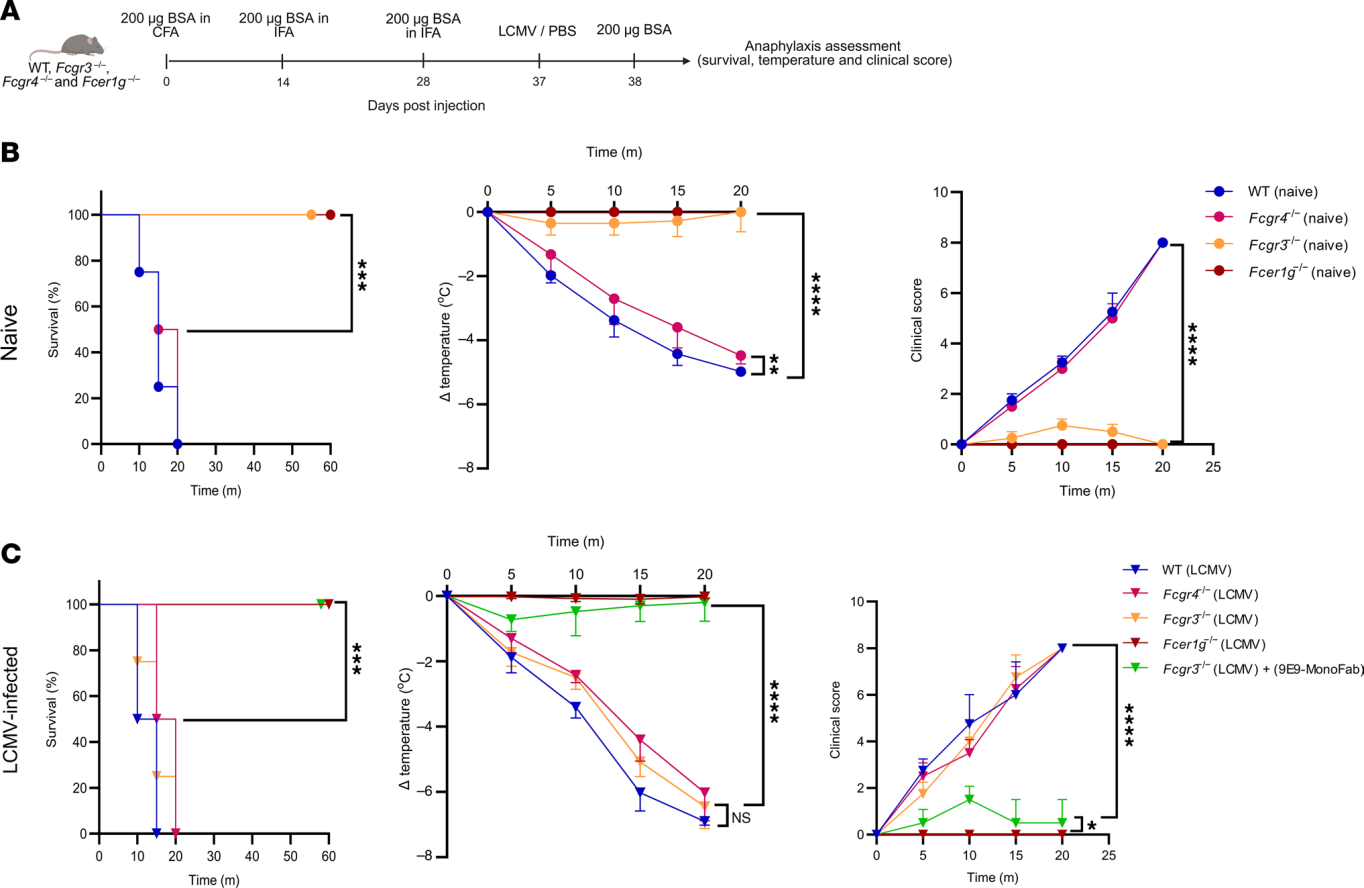
Viral infection shifts FcγR dependency toward FcγRIV in ASA. (**A**) Schematic representation of the ASA model used in **B**. WT, *Fcgr3^–/–^*, *Fcgr4^–/–^*, and *Fcer1g^–/–^* mice were immunized with BSA in CFA/IFA, boosted, and either infected with LCMV or treated with PBS as a control. Anaphylaxis was induced by i.v. injection of 200 μg BSA, and survival, body temperature, and clinical score (Supplemental Table 1) were monitored. (**B** and **C**) Survival, body temperature, and clinical score in naive (**B**) and LCMV-infected (**C**) WT, *Fcgr3^–/–^*, *Fcgr4^–/–^*, and *Fcer1g^–/–^* mice following BSA challenge. One group of infected *Fcgr3^–/–^* mice was additionally treated with the FcγRIV-blocking MonoFab 9E9 antibody. Data are from 1 experiment (*n =* 4 mice/group). All data are presented as the mean ± SD. ***P < 0.001 and ****P < 0.0001, by 2-way ANOVA (**B** and **C**) or log-rank test (**B** and **C**).

Given that the passive model may not fully reflect endogenous antibody generation, we next tested an active systemic anaphylaxis (ASA) model as previously described (23) (Figure 10A). Under naive conditions, *Fcgr3^–/–^* and *Fcer1g^–/–^* mice were completely protected, whereas WT and *Fcgr4^–/–^* animals developed pronounced anaphylactic symptoms (Figure 10B), consistent with a contribution of FcγRIII under steady-state conditions (12). Strikingly, infected *Fcgr3^–/–^* animals lost this protection and became highly susceptible, developing hypothermia and clinical scores comparable to those of infected WT mice (Figure 10C). As in the passive model, FcγRIV was indispensable, since FcγRIV blockade with MonoFab rescued *Fcgr3^–/–^* mice from infection-induced anaphylaxis. In contrast, *Fcer1g^–/–^* mice remained fully protected under both naive and infected conditions, confirming the requirement for activating FcγRs (FcγRI, FcγRIII and FcγRIV) (Figure 10C).

To define the effector compartment during ASA in infected animals, we depleted inflammatory monocytes during infection. Inflammatory monocyte depletion provided partial protection (~50% survival), whereas all infected *Fcgr3^–/–^* mice without depletion died (Supplemental Figure 10A).

These results demonstrate that viral infection profoundly enhanced susceptibility to systemic anaphylaxis and shifts the FcγRs requirement from FcγRIII under steady-state conditions to a dominant reliance on both FcγRIII and FcγRIV, with inflammatory monocytes serving as a key effector population under infection-associated immune priming.

## Discussion

Our findings identify FcγRIV as a pivotal driver of systemic anaphylaxis in our experimental models of viral infection, with IFN-I signaling serving as an essential upstream regulator. Using LCMV, VSV, HSV-1, and influenza infection models, we demonstrate that FcγRIV-dependent anaphylaxis represents a generalized, virus-enhanced phenomenon in which inflammatory monocytes play a central role. Importantly, IFN-I signaling markedly upregulated FcγRIV expression on these cells, thereby amplifying susceptibility to anaphylaxis. Our data do not indicate that viral infection alone triggered anaphylaxis; rather, infection created an immune milieu that enhanced sensitivity to IgG-mediated hypersensitivity.

Anaphylaxis is a rapid and life-threatening systemic hypersensitivity reaction. While our study focuses on its mechanisms during viral infection, similar dysregulation of immune pathways may exacerbate nonanaphylactic allergic reactions or localized anaphylactic reactions. The FcγRIV/PAF axis identified here could therefore in principle act as a general amplifier of allergic inflammation, even below the threshold of full anaphylaxis. This notion is supported by evidence that viral infections can exacerbate localized anaphylactic diseases such as asthma, largely through enhanced cytokine production and Th2-driven inflammation (33, 34). However, the contribution of FcγRs in this context is not well characterized. Our findings extend these observations by suggesting that infection-induced activation of FcγRs may contribute to a continuum of allergic severity, ranging from mild exacerbations to systemic anaphylaxis.

Several cell types have been involved in nonclassical anaphylaxis (30). Here, we further identified inflammatory monocytes as a key effector cell population mediating FcγRIV-dependent anaphylaxis during viral infection. Depletion strategies that substantially reduced this subset markedly attenuated both anaphylactic symptoms and the associated hypothermic response, whereas removal of neutrophils or RPMs had no effect. These data highlight the central role of inflammatory monocytes in FcγR-mediated hypersensitivity, extending prior observations that implicated these cells in antibody-dependent inflammation (12, 35). At the same time, CCR2-based approaches also affect other CCR2^+^ myeloid cell populations and do not fully eliminate inflammatory monocytes, which underscores that our conclusions are based on convergent, rather than exclusively monocyte-specific, evidence. Moreover, the absence of FcγRIV upregulation in *Ifnar1^–/–^* mice underscores and supports the mechanistic link between IFN-I signaling and FcγR expression, revealing an integrated regulatory axis that coordinates innate effector activation during infection.

Notably, our results uncover a strong dependence of FcγRIV expression on IFN-I signaling, complementing earlier studies that described IFN-γ–mediated modulation of FcγR expression (36, 37). Moreover, *Ifnar1^–/–^* mice, which lack IFN-I signaling, were completely protected from anaphylaxis, emphasizing the centrality of IFN-I in this process. However, these results stand in contrast to previous studies suggesting that IFN-I signaling is protective in classical anaphylaxis by limiting mast cell–mediated responses through the regulation of secretory granule homeostasis (38). Such discrepancies are critical for advancing our understanding of disease pathogenesis.

Translationally, we observed a related pattern in human monocytes during acute COVID-19, characterized by elevated frequencies of CD16^+^ (FcγRIIIa^+^) inflammatory subsets, which resemble FcγRIV upregulation in murine monocytes. Human FcγRIIIa (CD16a) is the functional homolog of murine FcγRIV (39), supporting the conceptual relevance of our findings across species. Furthermore, it is well established that human monocyte subsets, including nonclassical and intermediate monocytes, originate from classical monocytes and that their increased frequencies are linked to acute viral infections (40). This supports the notion that the elevated CD16-expressing monocytes observed in patients with COVID-19 likely represent a dynamic shift in monocyte differentiation triggered by the infection. Additionally, the cytokine storm observed in our study (Figure 2) aligns with previous reports showing that inflammatory monocytes are elevated and correlate with disease severity in patients with COVID-19, including those requiring ICU care (41). Likewise, infection of K18-hACE2 mice with SARS-CoV-2 recapitulated the activation pattern observed in LCMV infection, reinforcing the notion that FcγR-driven inflammatory monocyte activation represents a conserved response to acute viral challenge at the level of immune programming, rather than indicating that these viruses share pathogenic mechanisms. In both models, monocytes and macrophages adopt an inflammatory phenotype characterized by upregulation of activation markers and participation in cytokine and immune effector responses (as seen in patients with COVID-19 and in infected mice). Prior work in both SARS-CoV-2 and LCMV infections supports this view, implicating inflammatory monocytes and macrophages in cytokine overproduction and immunopathology (42, 43). Together, these data are consistent with a model in which acute viral infection primes monocytes and macrophages through FcγR upregulation and IFN-I–dependent signaling, thereby lowering the activation threshold for both immunopathology and FcγR-mediated hypersensitivity.

The dose-dependent relationship between IFN-I levels and anaphylaxis susceptibility further underscores the quantitative influence of IFN-I on FcγRIV-driven effector responses. This finding is particularly relevant for type I interferonopathies such as systemic lupus erythematosus (SLE), in which heightened IFN-I activity contributes to FcγR-mediated immune activation (20, 44). Infection can exacerbate these pathways; indeed, patients with SLE experience a significantly higher rate of disease flares following SARS-CoV-2 infection (17.6% vs. 5.5%), independent of classical serological markers (45). These observations support a model in which infection-induced IFN-I responses lower the activation threshold of FcγR-bearing effector cells, thereby enhancing inflammatory and autoimmune manifestations.

Our findings establish PAF as a critical mediator of FcγRIV-driven anaphylaxis during viral infections. Elevated serum PAF levels were observed exclusively in infected mice following FcγRIV crosslinking, and pharmacological blockade of the PAF-R completely protected against anaphylactic symptoms and mortality. These findings are consistent with prior reports highlighting the role of PAF in nonclassical anaphylaxis (12, 30). The dependence of PAF production on both IFN-I signaling and inflammatory monocytes further underscores the integrated nature of these pathways in driving FcγRs-mediated responses.

Our study also provides mechanistic insight into how viral infections may predispose to anaphylaxis and allergic disease through FcγR upregulation on inflammatory monocytes. Translating these findings to human diseases requires careful consideration, but epidemiological data support this connection. A multinational cohort study from South Korea, Japan, and the United Kingdom reported that individuals recovering from COVID-19 had a significantly increased risk of developing new-onset allergic diseases, including asthma and allergic rhinitis, beyond 30 days after infection, with a higher risk among those with severe COVID-19, and a protective effect of vaccination (46). Additionally, respiratory viral infections such as rhinovirus and respiratory syncytial virus (RSV) are major triggers of asthma exacerbations, accounting for 50%–80% of adult cases (47). These clinical observations are compatible with our mechanistic findings, suggesting that infection-induced FcγR upregulation and IFN-I–driven activation of monocytes may amplify allergic responses and potentially promote severe reactions. While these effects may not always reach the threshold of anaphylaxis, they could potentiate preexisting allergic tendencies, consistent with reports of new-onset allergic diseases after COVID-19 (46). Together, these data underscore the need for further research to elucidate the role of viral infections in the pathogenesis of anaphylaxis and other allergic diseases.

Finally, we demonstrate that MonoFab, a monovalent antibody targeting FcγRIV, effectively and prophylactically prevented virus-induced anaphylaxis, reducing both mortality and PAF levels. These findings highlight FcγRs blockade as a potential therapeutic strategy for managing virus-induced hypersensitivity and support further evaluation of FcγR-targeted approaches in settings of heightened IFN-I activity, extending earlier reports describing FcγR-targeted therapies for autoimmune diseases (48).

Collectively, our study delineates a mechanistic pathway linking viral infection, IFN-I signaling, FcγRIV upregulation, and PAF-mediated anaphylaxis. These results identify inflammatory monocytes as central contributors to FcγR-driven hypersensitivity and provide translational insights relevant to both acute viral infection and chronic inflammatory disease. Future investigations should explore the potential of FcγR blockade or modulation of IFN-I responses as therapeutic avenues to mitigate infection-associated allergic and anaphylactic complications.

### Study limitations.

While this study provides valuable insights into the mechanisms of FcγR-mediated hypersensitivity reactions during viral infections, several limitations should be acknowledged. First, the findings in murine models may not fully recapitulate the complexity of human immune responses, despite the observed parallels, such as the increased frequencies of CD16-expressing monocytes in patients with COVID-19. Second, the study focuses primarily on FcγRIV in mice, and while FcγRIIIa (CD16a) serves as its homolog in humans, further investigations are needed to confirm whether similar FcγRIIIa-mediated mechanisms drive anaphylaxis in humans. While these data demonstrate that IFN-I signaling is required for FcγRIV upregulation in vivo, IFN-I alone may not fully reproduce the broader infection-associated changes or fully explain all aspects of susceptibility.

In addition, although 9E9-mediated FcγRIV crosslinking represents an artificial activation method, we performed complementary proof-of-principle experiments using a BSA–anti-BSA immune complex model in which FcγRs were engaged through endogenous IgG immune complexes. These limited studies produced qualitatively similar results, supporting the relevance of the FcγRIV-dependent mechanism, although this model was not explored in depth. We also acknowledge that CCR2-based depletion affects multiple CCR2^+^ myeloid subsets, that FcγR expression differs across cell types and species, and that LCMV serves as a model for robust IFN-I induction rather than for SARS-CoV-2 pathogenesis. Together, these considerations define the scope and boundaries of our conclusions.

Finally, certain limitations relate to the human data included in this study: the patient cohort had a different mean age compared with the healthy control group, which may have introduced confounding factors related to age-associated immune changes. Moreover, the gating strategy used for analysis of monocytes from human donors was limited, potentially restricting the resolution of monocyte subsets and their detailed characterization. However, earlier studies have reported similar observations, supporting the robustness of our findings despite these limitations. In addition, our human data are observational and do not establish a causal link between viral infection, FcγR modulation, and clinical anaphylaxis. These limitations highlight the need for further studies to validate and expand upon our findings in diverse clinical contexts.

## Methods

### Sex as a biological variable

Both male and female mice and humans were included in this study. Experimental groups were balanced for sex where possible. However, sex was not considered as a biological variable, and data from males and females were pooled for analysis.

### Mice

This study utilized mice sourced from various strains, including C57BL/6 (WT, JAX: 00664) obtained from The Jackson Laboratory and bred in-house. *Fcgr3^–/–^* and *Fcgr4^–/–^* were acquired from Falk Nimmerjahn (Friedrich-Alexander-Universität Erlangen-Nürnberg [FAU], Germany). *Vav1*-Cre^Tg^ *Spic^fl/fl^* mice were acquired from Manfred Kopf (Institute of Molecular Health Sciences, Department of Biology, ETH Zürich, Zürich, Switzerland) via Wiebke Hansen. Triple-KO mice (*MyD88^–/–^* *Trif^–/–^* *Cardif^–/–^*) and *Ifnar1^fl/fl^* *Cx3cr1*-CreER^tg/+^ and littermate controls were acquired from Ulrich Kalinke (TWINCORE, Hannover, Germany). *Ifng^–/–^* (JAX:002287), *Fcer1g*^−/−^ (JAX: 002847), *Ifnar1^–/–^* (JAX: 028288), and *Kit^W-sh^* (Sash-KO; JAX:030764) mice were acquired from The Jackson Laboratory. Mice used in this study were maintained on a C57BL/6 genetic background, with back-crossing performed at least 10 times when necessary. Male and female mice aged 8–12 weeks were used for most experiments, while mice aged 14–20 weeks were used for the active anaphylaxis model. K18 [B6.Cg-Tg(K18-ACE2)2Prlmn/J] mice were provided by Thomas Gramberg (Harald zur Hausen Institute of Virology, Friedrich-Alexander-Universität Erlangen-Nürnberg, Erlangen, Germany). To ensure unbiased allocation, age- and sex-matched animals were randomly assigned to treatment groups. All mice were housed in individually ventilated cages under specific pathogen–free conditions.

### Human participants

Peripheral blood samples were collected from 64 patients with COVID-19 (24 females and 40 males) and 37 healthy volunteers (22 females and 15 males) between February 2021 and May 2021, following the approval of the study protocol (approval number: 21-9883-BO). The patients with COVID-19 had an average age of 60 years and exhibited mild-to-moderate symptoms requiring hospitalization but not intensive care or intubation. The healthy volunteers were colleagues from the University Hospital Essen and had an average age of 35 years. The analysis of whole blood samples from patients and healthy donors was conducted at the Institute of Immunology, Medical Faculty at the University Duisburg-Essen. Blood samples were transported safely from the Department of Clinical Medicine to the Institute of Immunology in accordance with established guidelines.

### In vivo virus infection

Mice were infected i.v. with LCMV-WE (1 × 10^6^ PFU), LCMV-docile (5 × 10^5^ PFU), VSV (1 × 10^8^ PFU), HSV-1 (1 × 10^6^ PFU), or influenza A virus (1 × 10^6^ PFU). LCMV strains (WE and docile) and VSV were originally provided by Rolf Zinkernagel (Institute of Experimental Immunology, ETH Zürich, Switzerland). The influenza A virus PR8-GLuc (H1N1, A/Puerto Rico/8/34) expressing *Gaussia* luciferase was provided by Matthias Tenbusch (Harald zur Hausen Institute of Virology, FAU Erlangen, Germany). HSV-1F was provided by Hartmut Hengel (Institute of Virology, University of Freiburg, Germany).

Virus stocks were propagated on BHK-21 cells (for VSV and LCMV strains; American Type Culture Collection [ATCC], CRL-8544), Vero cells (for HSV-1; ATCC, CCL-81), or MDCK II cells (for influenza A virus; ATCC, CRL-2936) at a MOI of 0.01 or 0.001, as appropriate. For the active anaphylaxis model, LCMV-WE was propagated on BHK-21 cells using serum-free medium.

K18 [B6.Cg-Tg(K18-ACE2)2Prlmn/J] mice (49 weeks old) were first anesthetized according to institutional guidelines, followed by intranasal infection with recombinant SARS-CoV-2 WT virus (49). For each mouse, 10,000 PFU virus were diluted in 30 μL sterile PBS and applied into 1 nostril. Upon infection, clinical scores and body weights of all mice were monitored daily. Forty-eight hours after infection, the animals were euthanized.

### Systemic anaphylaxis

#### Passive systemic anaphylaxis.

Sera were collected from BSA-immunized mice on day 37, following an immunization schedule consisting of 2 BSA (catalog 126579-100GM, MilliporeSigma) immunizations in CFA (catalog vac-cfa-10, InvivoGen) on days 0 and 14, and a single BSA immunization in incomplete Freund’s adjuvant (IFA) (catalog vac-ifa-10, InvivoGen) on day 30. IgG was purified from the sera using a protein G sepharose column (affinity chromatography). Purified IgG (1,000 μg) was then i.v. injected into mice, which were challenged with 300 μg BSA via i.v. administration 3 hours later. In a separate set of experiments, anaphylaxis was induced by injecting mice with anti–mouse CD16.2 (clone 9E9) antibody (catalog C859, Leinco Technologies).

#### Active systemic anaphylaxis.

Mice were actively immunized with BSA according to the following schedule (23): 200 μg BSA emulsified in CFA was administered on day 0, followed by 200 μg BSA in IFA on days 14 and 28. On day 30, BSA-specific serum IgG2a titers were determined by ELISA. Mice with comparable IgG2a titers were grouped for challenge. On day 37, mice were infected i.v. with LCMV-WE or given PBS to serve as naive controls. Twenty-four hours after infection, mice were challenged i.v. with 200 μg BSA, and systemic anaphylaxis severity was scored.

Central body temperature was recorded using a digital thermometer as previously reported (50). Clinical scores were assessed according to the criteria outlined in Supplemental Table 1. To avoid personal bias, clinical evaluations were performed by an independent observer who was blinded to the experimental groups. Mice that reached a clinical score of 8 were defined as having reached the humane endpoint and were euthanized for statistical analysis. For technical reasons, in some experiments, body temperature was recorded within 10 minutes of reaching this score, immediately before euthanasia.

### Cell depletion and receptor blocking

The depletion antibodies utilized were anti-Ly6G (clone 1A8) and anti–GR-1 (clone RB6-8C5) — all procured from Bio X Cell — and each was administered i.p. at a dosage of 200 μg per mouse. Additionally, anti-CCR2 (clone MC21) was obtained from Matthias Mack and administered i.v. at a dosage of 35 μg per mouse. For IFN-I receptor blockade, mice received 1,000 μg anti-IFNAR1 antibody (clone MAR1-5A3, Bio X Cell) i.p. 5 hours prior to infection. Monocyte and macrophage depletion was achieved by administering 200 μL clodronate per mouse. For preparation of 9E9-MonoFab, the Pierce Fab Preparation Kit (catalog 44985, Thermo Fisher Scientific) was used according to the manufacturer’s instructions. For blocking of the PAF-R, 100 μg WEB2086 (catalog SML0238, MilliporeSigma) was administrated i.v. 20 minutes before 9E9 administration.

### Tamoxifen induction in vivo

To induce Cre recombinase activity, *Ifnar1^fl/fl^* *Cx3cr1*-CreER^tg/+^ and littermate control *Ifnar1^fl/fl^* *Cx3cr1*-CreER^+/+^ mice received i.p. injections of tamoxifen for 5 consecutive days (3 mg/ mouse; MilliporeSigma, T5648). Tamoxifen was dissolved in 90% corn oil (Thermo Fisher Scientific, 405435000) and 10 % ethanol. Mice were kept for 48 hours after the final injection before infection with LCMV-WE (1 × 10^6^ PFU).

### scRNA-Seq data analysis

Publicly available scRNA-Seq datasets from the NCBI’s Gene Expression Omnibus (GEO) database were obtained through the CZ CELLxGENE Collection Portal. The analysis included 5 datasets of PBMCs from healthy donors and patients with COVID-19 with different disease severities (GSE150728, GSE150861, GSE155673, GSE149689, and GSE152418). Quality control and sample integration, as described by the resource providers, was confirmed. Only cells annotated as classical monocytes were retained for downstream analysis. Monocytic identity was verified by expression of canonical markers (CD14, S100A8, S100A9, CCR2, HLA-DRA). FCGR3A expression was compared between groups using the Kruskal-Wallis test with post hoc pairwise Wilcoxon rank-sum tests. The *P* values obtained were corrected for multiple comparisons using the Benjamini-Hochberg procedure. Cells with non-zero expression of both CD14 and FCGR3A were annotated as double-positive. Counts of double-positive cells were compared using a χ^2^ test with post hoc pairwise Fisher’s exact tests.

### In vitro cell isolation and treatment

Bone marrow was harvested from the femurs and tibias of WT and *Ifnar1^−/−^* mice, and RBCs were lysed using RBC lysis buffer (PAN Biotech, catalog P10-90100). The remaining cells were collected by centrifugation, and monocytes were isolated using magnetic-activated cell sorting (MACS) (Miltenyi Biotec, catalog 130-100-629). The isolated monocytes were resuspended at a density of 1 × 10^6^ cells/mL in complete DMEM supplemented with 20 ng/mL macrophage CSF (M-CSF) (PeproTech). Cells were treated with varying concentrations of IFN-β (51), while untreated cells served as controls. Cultures were incubated at 37°C with 5% CO_2_ for 18 hours prior to flow cytometry staining.

### Histology

Histological analysis of snap-frozen splenic tissues was performed using staining with monoclonal antibodies, all diluted at 1:100. Briefly, organs were embedded in Tissue-Tek (Sakura Finetek) and snap-frozen in liquid nitrogen. Frozen tissues were sectioned into 8.0 μm thick slices, air-dried, and fixed in acetone for 10 minutes. Slides were then incubated in blocking buffer (PBS with 4% FBS) for 25 minutes at room temperature to minimize nonspecific binding. Antibody mixtures were prepared in PBS with 4% FBS and incubated with the tissue sections for 45 minutes at room temperature. Between staining steps, slides were washed twice with PBS. Antibodies were then mixed and applied at the specified ratios. After final washing steps, 1 drop of fluorescence mounting media was added to each slide, and coverslips were applied. Slides were dried in the dark for at least 30 minutes before image acquisition. Images were captured using a Keyence BZ-9000 microscope (Keyence).

### Cytokine measurement

Serum cytokines were analyzed with the LEGENDplex Mouse Anti-Virus Response Panel (13-plex) with a V-bottom Plate (BioLegend, catalog 740622) according to the instructions and recommendation of the manufacturer. PAF was measured from serum of mice using a commercial ELISA kit (Abbexa, catalog ABX254319) according to the manufacturer’s instructions.

### Statistics

Data are expressed as the mean ± SD. For comparisons between 2 groups, 2-tailed, unpaired Student’s *t* tests were used unless otherwise specified. For comparisons involving more than 2 groups, 1-way ANOVA was applied. Two-way ANOVA was used for analyses incorporating 2 independent variables, such as time-course experiments or genotype × treatment interactions unless otherwise mentioned. A *P* value of less than 0.05 was considered significant.

### Study approval

Studies involving humans were reviewed and approved by the IRB of the University Hospital Essen (Essen, Germany). All participants provided written informed consent prior to enrollment, in accordance with the Declaration of Helsinki. All animal experiments were conducted with authorization from the Landesamt für Natur, Umwelt und Verbraucherschutz Nordrhein-Westfalen (LANUV NRW, Recklinghausen, Germany) and in compliance with the German Animal Protection Law. Institutional guidelines at the Ontario Cancer Institute of the University Health Network and McGill University were also strictly followed.

### Data availability

All underlying numerical data used to generate the graphs in this study are provided in the accompanying Supporting Data Values file. Reagents used in the study are listed in Supplemental Table 2. Additional datasets, including raw flow cytometry files, imaging data, and processed analysis outputs, are available from the corresponding author upon reasonable request. Human participant data have been deidentified in accordance with institutional ethics guidelines; because of privacy protections, individual-level raw data cannot be publicly deposited but can be shared in anonymized form upon request and pending institutional approval.

## Author contributions

AE designed and conceptualized the experiments. AE and HA wrote the manuscript. AE, HA, JS, SD, JF, GME, TCC, EW, JFK, AR, and FK performed the experiments. AE, KSL, HA, FN, AS, and NBL analyzed and interpreted the data. MM, FN, MK, KS, WH, TG, and UK provided material and technical details for the study. KSL supervised the project, provided conceptual input, and acquired funding. All authors critically revised and approved the manuscript.

## Funding support

- German Research Foundation (DFG) through the Research Training Groups RTG1949 and RTG2098 and grant LA1419/10-1.
- University of Duisburg-Essen.
- German Research Foundation (CRC1755-P3 and TRR369-C01) to FN.
- Interdisciplinary Center for Clinical Research (IZKF), University Hospital Erlangen to FN.

## Supplementary Material

Supplemental data

Supporting data values

## Version 1 02/16/2026

Electronic publication
