# Supplementary material for: Type I IFN–dependent FcγRIV signaling in murine monocytes promotes lethal anaphylaxis during viral infections
Source: J Clin Invest. 2026 Feb 16;136(4):e192371. doi: 10.1172/JCI192371 (PMC12904709; doi:10.1172/JCI192371)
Supplement: Supplemental data [file jci-136-192371-s216.pdf]

Supplemental Figure 1. Validation and characterization of the FcγRIV-mediated anaphylaxis model

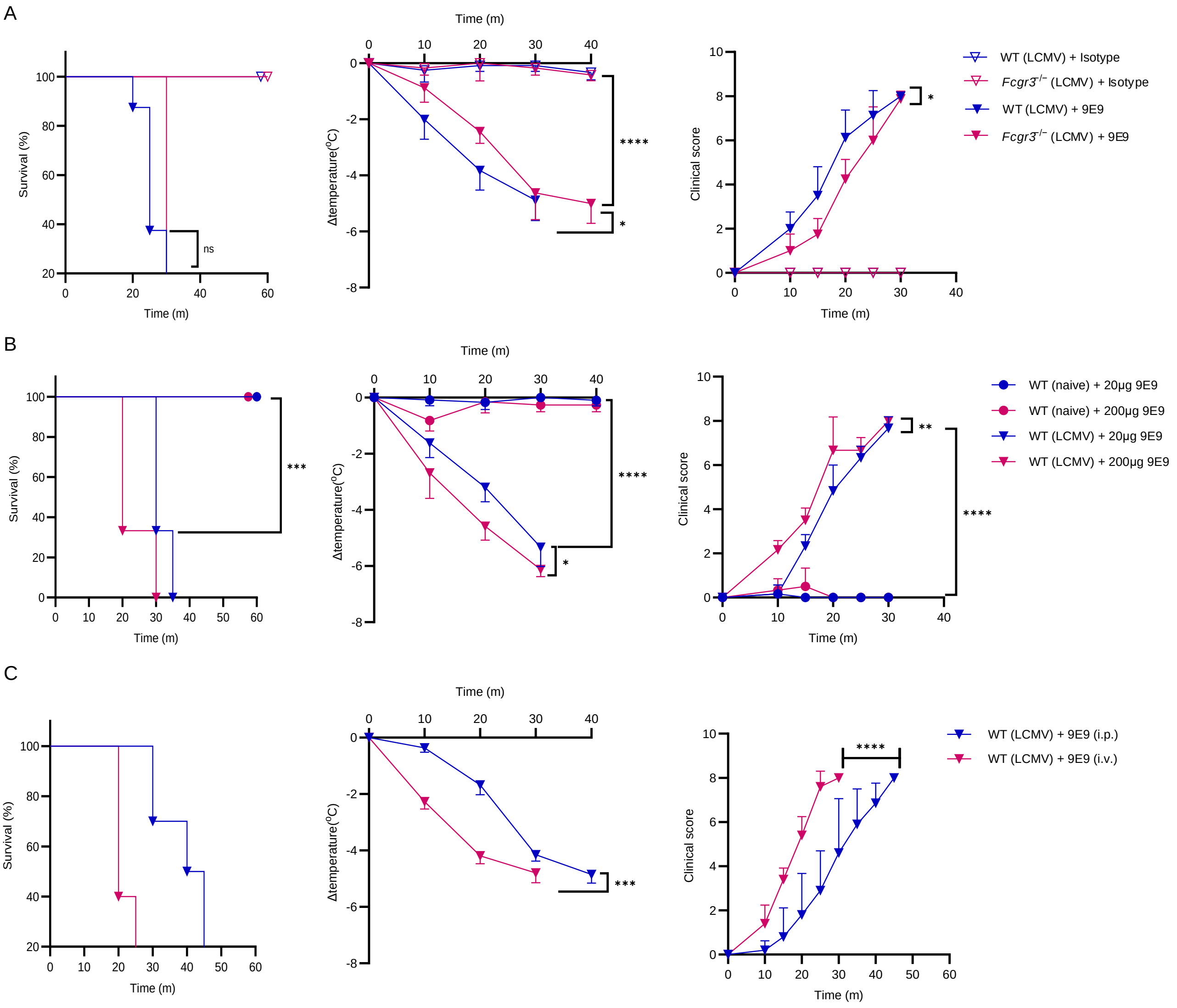

Supplemental Figure 2. IFN-I signaling determines susceptibility to FcγRIV-mediated anaphylaxis across viral infections

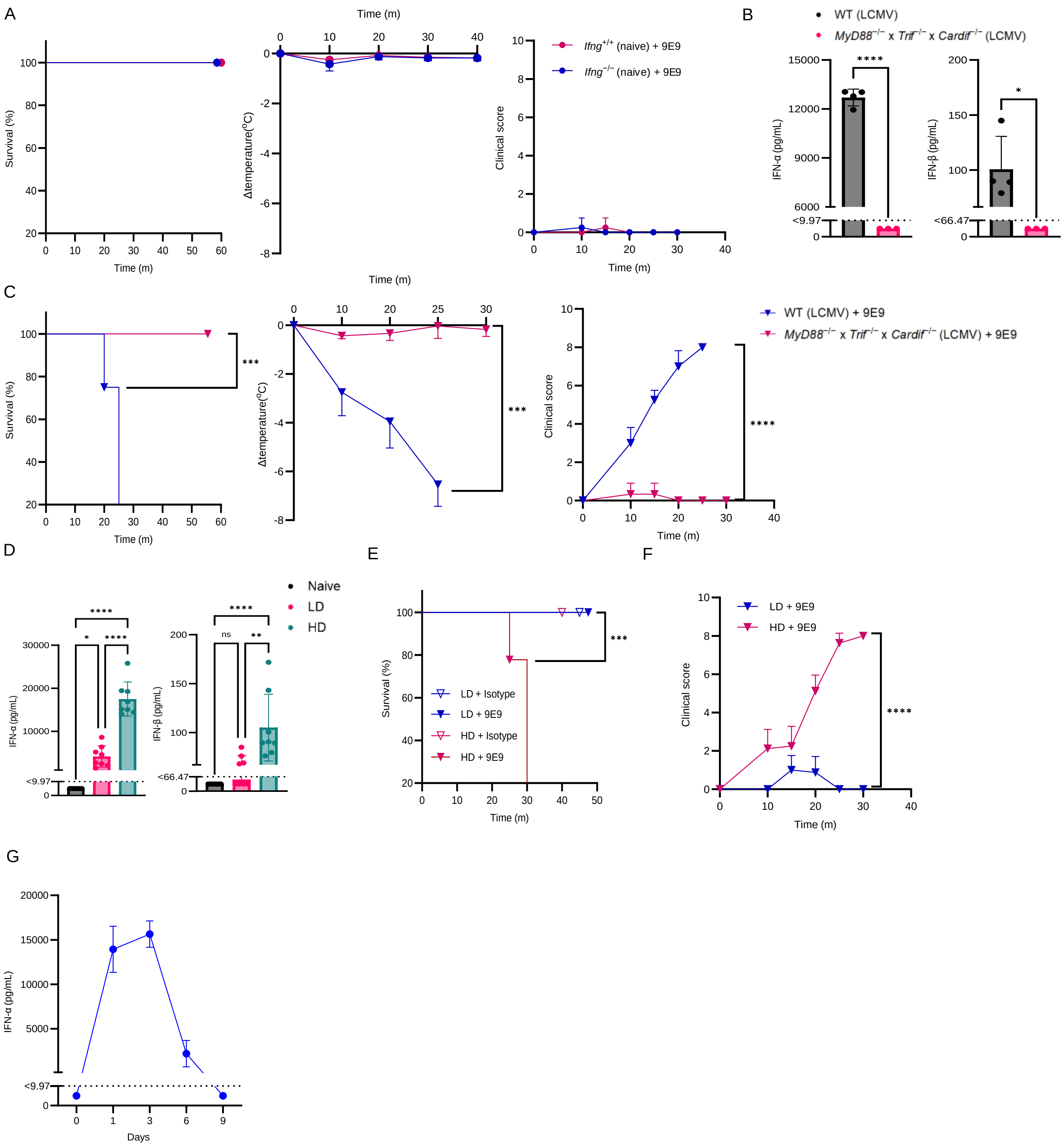

Supplemental Figure 3. IFN-I regulates FcγRIV expression across myeloid cell subsets

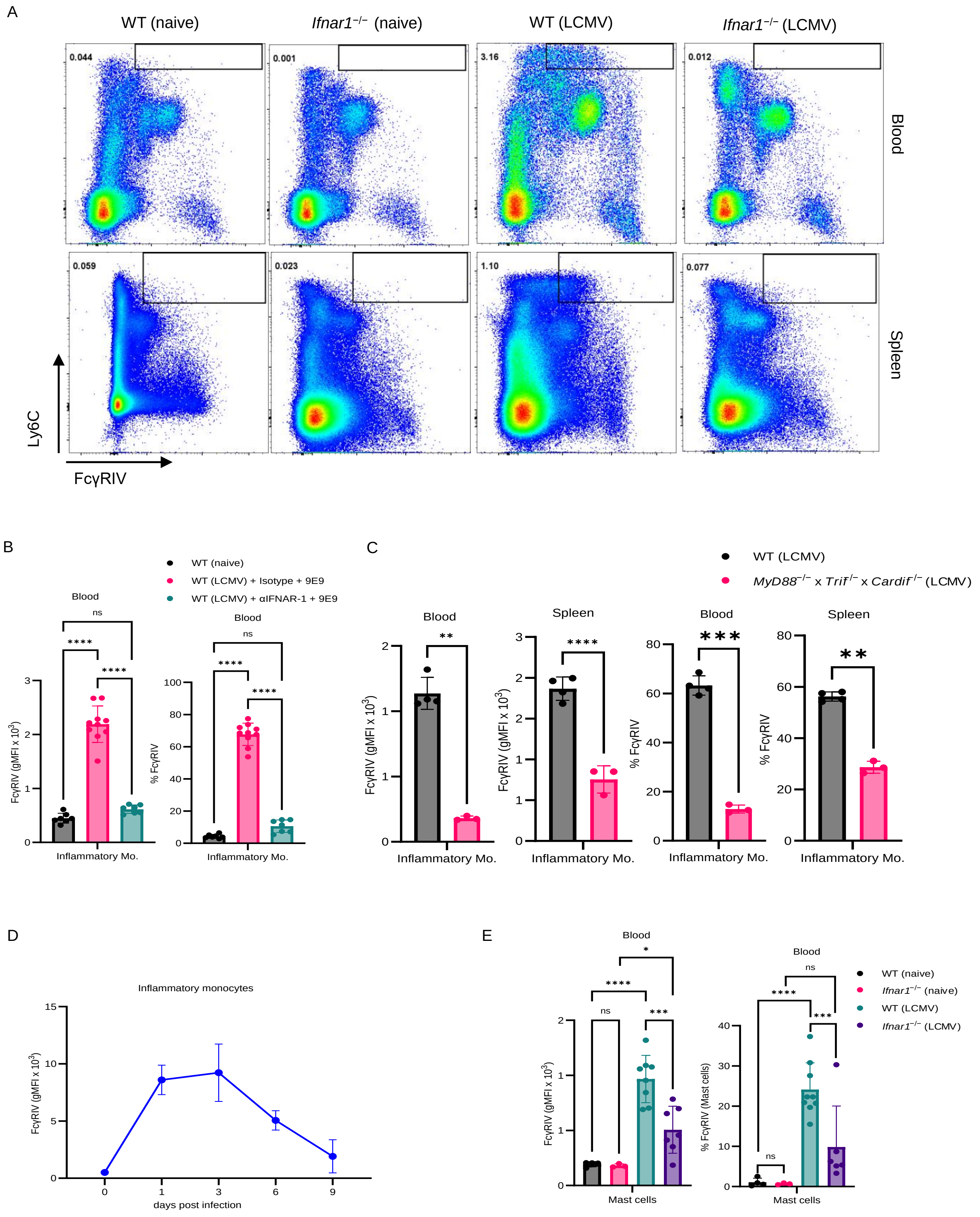

Supplemental Figure 4. Neutrophils and mast cells are dispensable for FcγRIV-mediated anaphylaxis

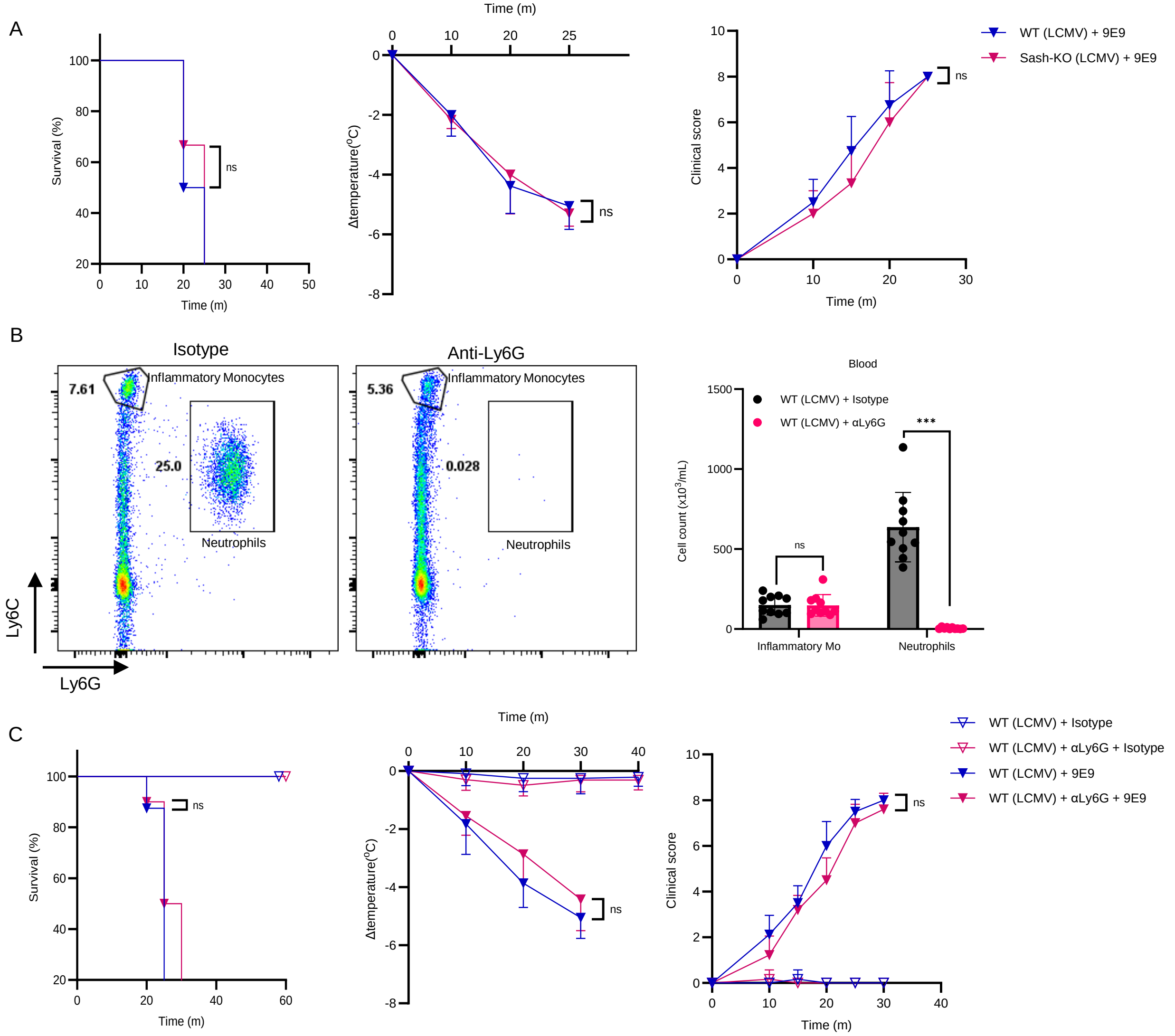

Supplemental Figure 5. FcγRIV expression on splenic macrophage subsets

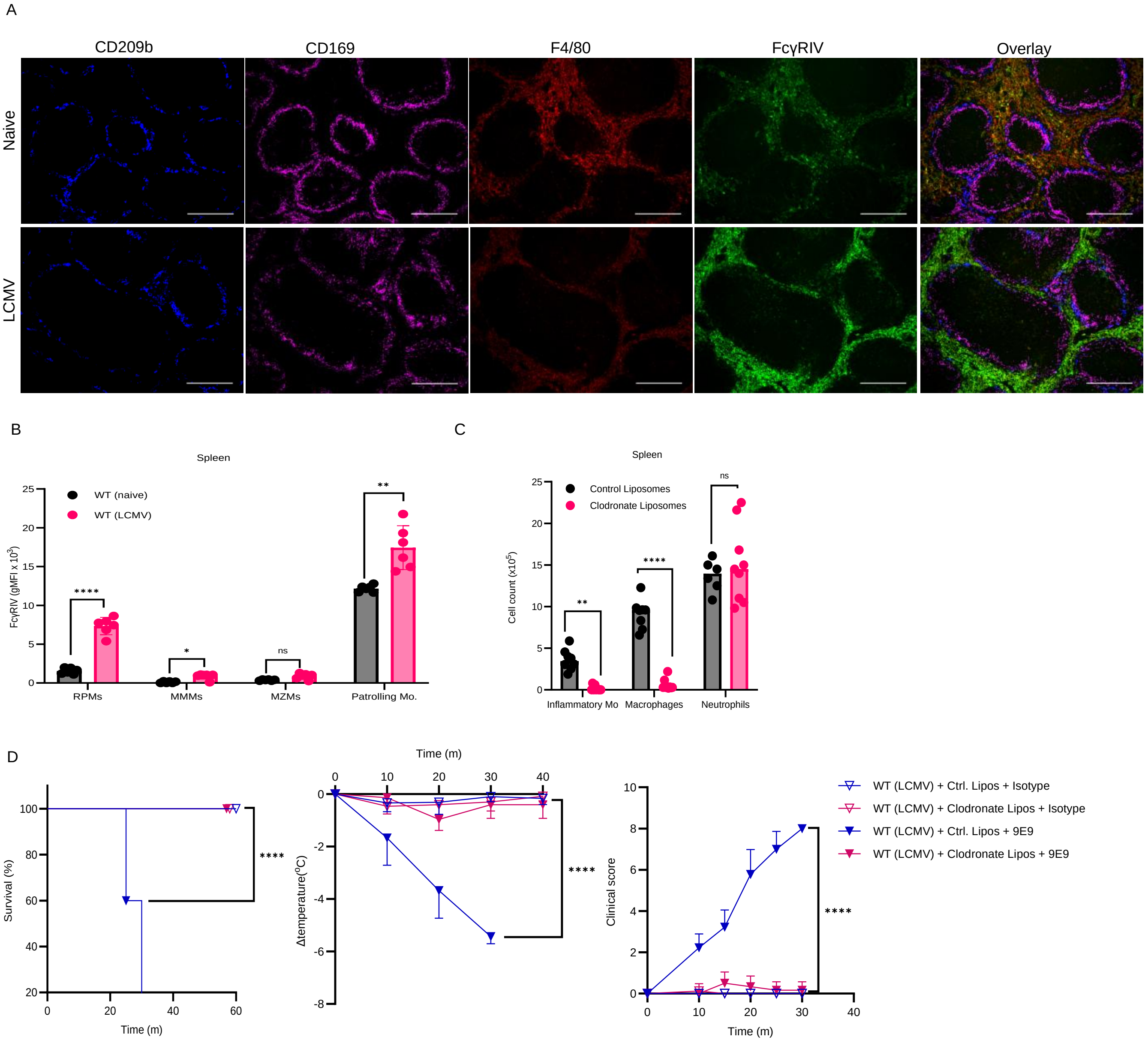

Supplemental Figure 6. Red pulp macrophages are dispensable for FcγRIV-dependent anaphylaxis

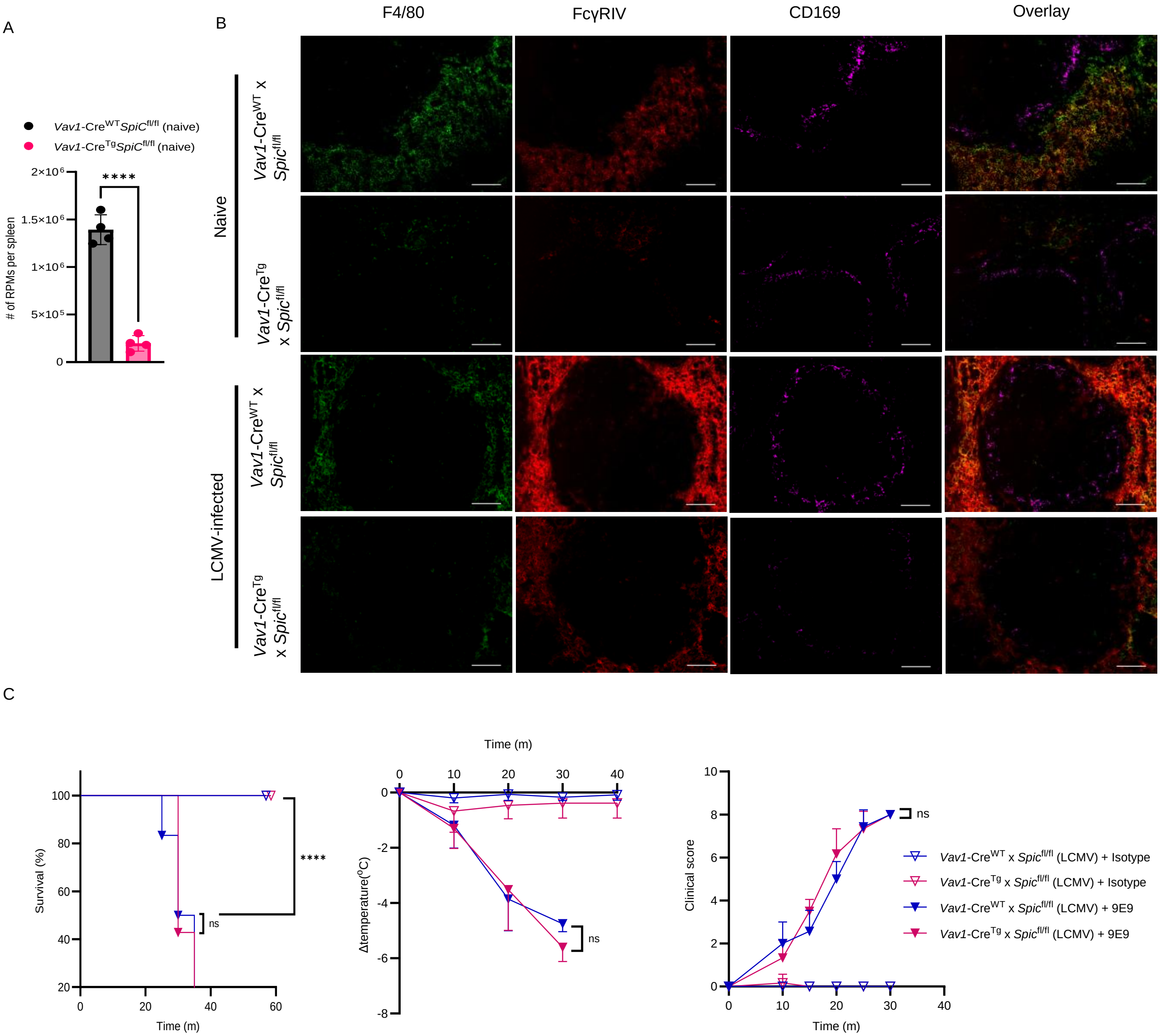

Supplemental Figure 7. Inflammatory monocytes are required for FcγRIV-driven anaphylaxis

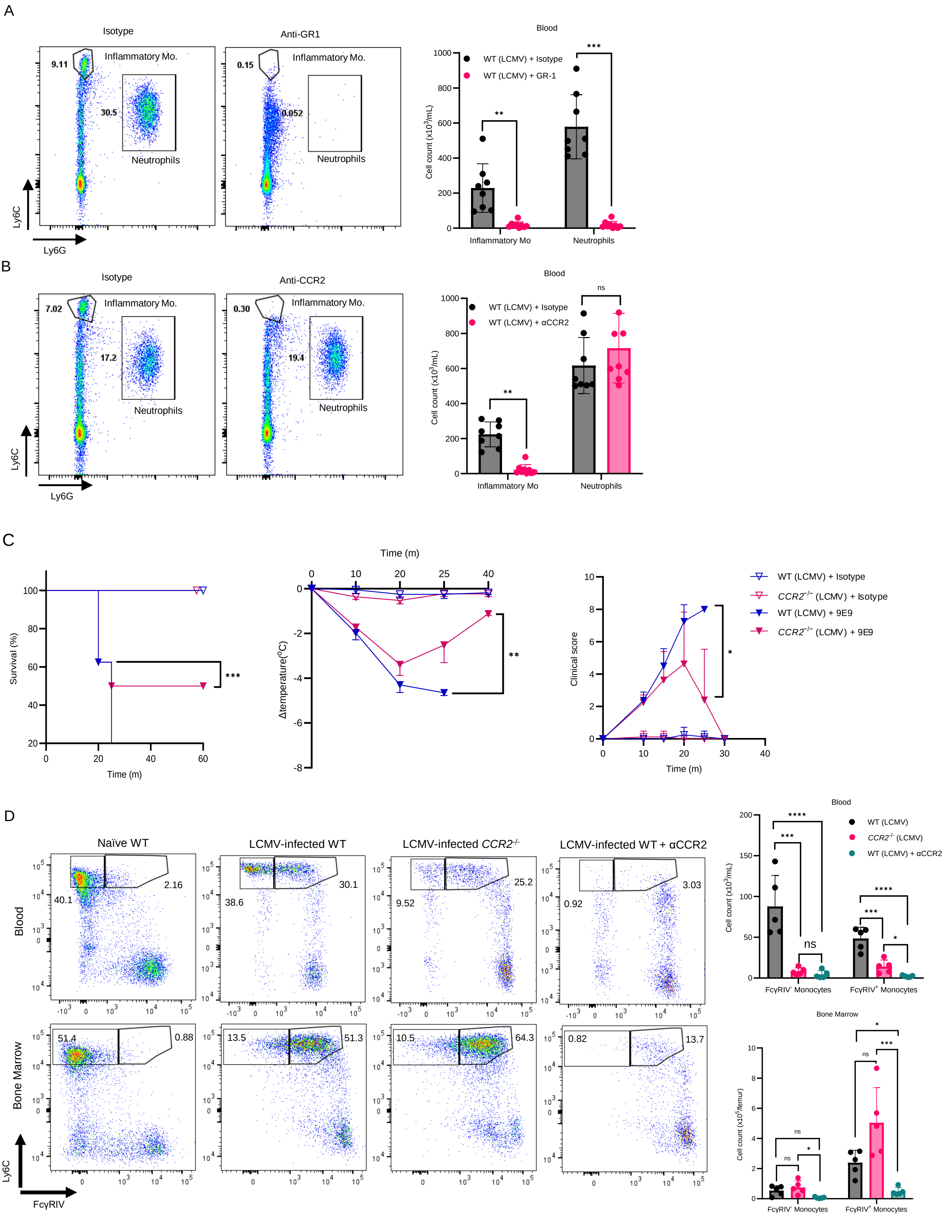

Supplemental Figure 8. Different viruses induce FcγRIV upregulation on inflammatory monocytes

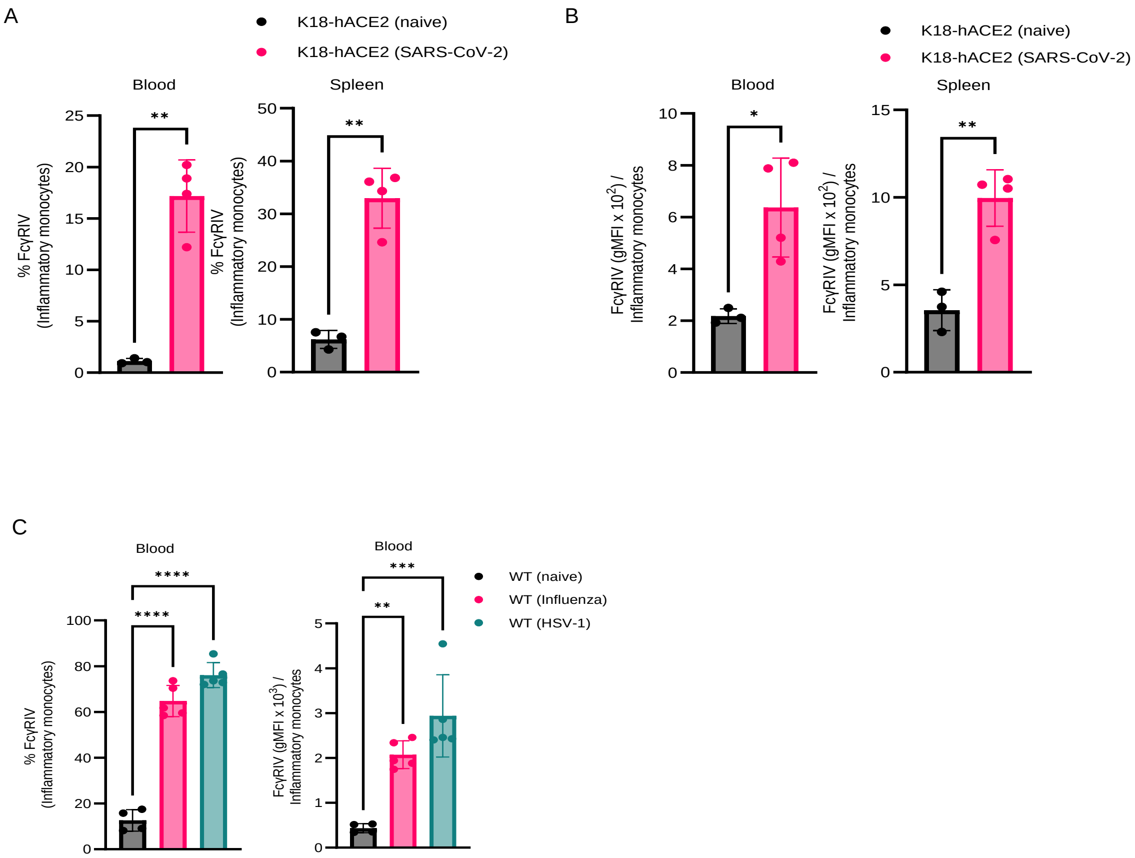

Supplemental Figure 9. Mast cells and FcγRIII are dispensable for PAF production upon FcγRIV activation

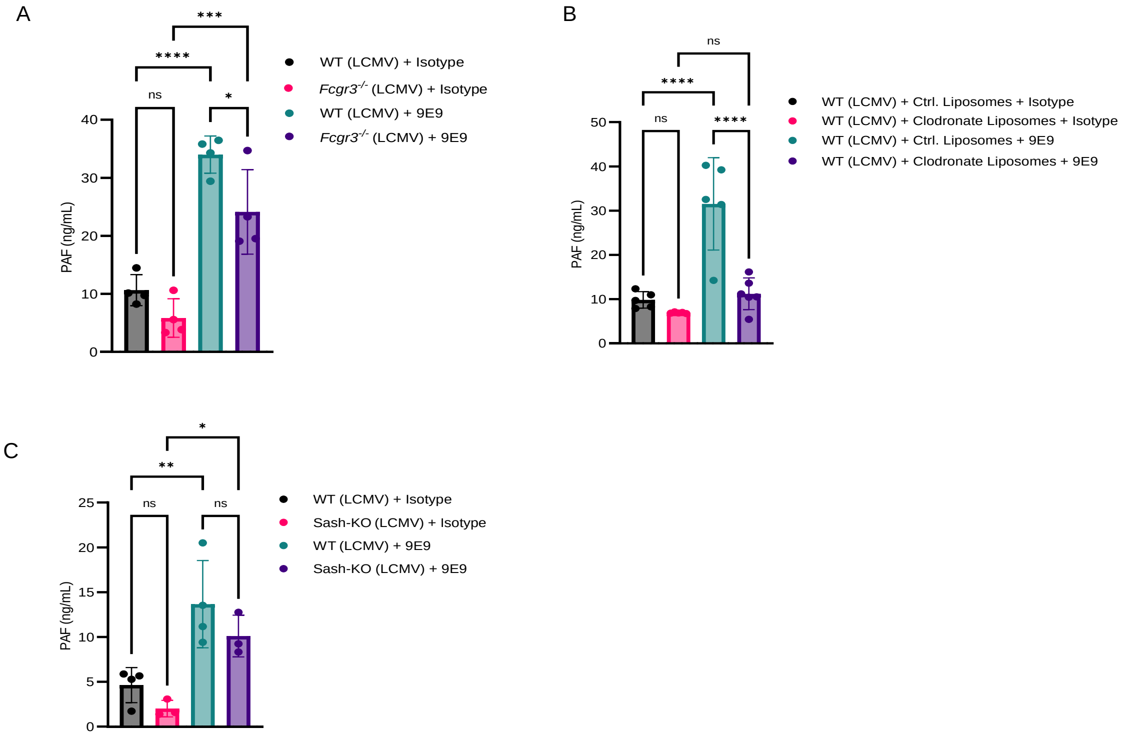

Supplemental Figure 10. Inflammatory monocytes are contributing to FcγRIV-dependent systematic active anaphylaxis after infection

A

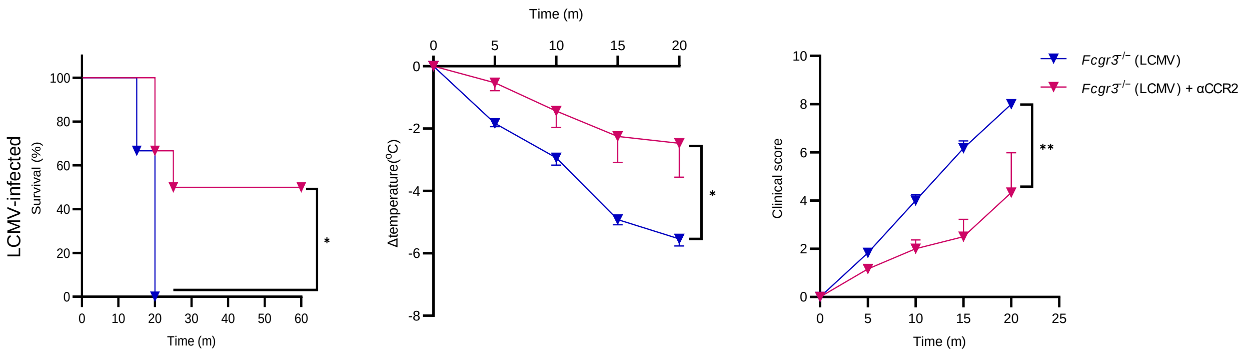

Supplemental Table 1. Clinical scoring system used for mice anaphylaxis

Attributes	Score
Hunched	0-2
Lack of movement	0-2
Lack of alertness	0-2
Anaphylactic grade[A]	0-2

[A]The anaphylactic grade is based on the comparison between the measured body temperature before and 30mins (Δt) after antigen challenge. Afterwards the anaphylactic grade for each mouse was sorted into one of the three following categories: Score 0 = "no shock": Δt ≤ -1, without mortality rate; Score 1 = "minor shock": Δt < -4, without mortality rate; Score 2 = "major shock": Δt ≥ -4, and/or mortality before 30mins

Supplemental Table 2. Table of reagents used in the study

Target	Catalog number	Clone name/ name	Source	Application
F4/80	53-4801-82	BM8	ThermoFischer	Histology
F4/80	47-4801-82	BM8	ThermoFischer	Flowcytometry
CD117 (c-Kit)	105818	2B8	BioLegend	Flowcytometry
CCR2	747966	475301	BD Biosciences	Flowcytometry
CD43	563206	S7	BD Biosciences	Flowcytometry
Cx3CR1	149014	SA011F11	BioLegend	Flowcytometry
CD11b	557857	M1/70	BD Biosciences	Flowcytometry
CD16.2	149521	9E9	BioLegend	Flowcytometry / Histology
Ly6C	45-5932-82	HK1.4	ThermoFischer	Flowcytometry
CD45.2	56-0454-82	104	ThermoFischer	Flowcytometry
Ly6G	11-9668-82	1A8	ThermoFischer	Flowcytometry
CD19	11-0193-85	1D3	ThermoFischer	Flowcytometry
CD3	11-0031-85	145-2C11	ThermoFischer	Flowcytometry
Ly6G	BE0075-1	1A8	BioXcell	Depletion
GR-1	BE0320	RB6-8C5	BioXcell	Depletion
CCR2	-	MC21	Prof. Matthias Mack	Depletion
ifnar1	BE0241	MAR1-5A3	BioXcell	Blocking
PAF receptor	SML0238	WEB2086	Sigma-Aldrich	Blocking
CD169 (Siglec-1)	130-124-896	REA197	Miltenyi Biotec	Flowcytometry
CD209	130-117-786	REA125	Miltenyi Biotec	Flowcytometry
CD8	300914	HIT8a	BioLegend	Flowcytometry
CD11b	557657	M1/70	BD Biosciences	Flowcytometry
CD16	563830	3G8	BD Biosciences	Flowcytometry
CD14	325604	HCD14	BioLegend	Flowcytometry
CD56	12-0567-42	CMSSB	ThermoFischer	Flowcytometry

Supplemental Figure 1. Validation and characterization of the FcγRIV-mediated anaphylaxis model

(A) WT and *Fcgr3*$^{-/-}$ mice were infected with LCMV (1×10^{6} PFU) and treated 24 h.p.i with 200 µg 9E9 or an isotype. Survival, body temperature and clinical score (Supplemental Table 1) were monitored over time. Results show the pooled data from two independent experiments (*n* = 4-5 mice/group/experiment).

(B) Naive and LCMV-infected WT mice (1×10^{6} PFU) were treated intravenously 24 h.p.i. with the 9E9 antibody at different concentrations (20 µg and 200 µg). Survival, body temperature, and clinical score were monitored over time. Results show the pooled data from two independent experiments with similar results (*n* = 3 mice/group/experiment).

(C) LCMV-infected WT mice (1×10^{6} PFU**)** were treated 24 h.p.i. with 9E9 (200 µg) i.v. or i.p. Survival, body temperature, clinical score were monitored over time. Results show the pooled data from two independent experiments (*n* = 5 mice/group/experiment).

All data are represented as mean ± SD. Statistical significance was determined using log-rank test for survival (A and B), two-way ANOVA for body temperature and clinical score (A and B). *p < 0.05, ***p < 0.001, ****p < 0.0001.

Supplemental Figure 2. IFN-I signaling determines susceptibility to FcγRIV-mediated anaphylaxis across viral infections

(A) Survival, body temperature, and clinical score of naive *Ifng*$^{+/+}$ and *Ifng*$^{-/-}$ mice treated with 9E9 (200 µg). Data from one experiment (*n* = 4 mice/group).

(B) Serum levels of IFN-α and IFN-β in LCMV-infected (1×10^{6} PFU) WT and *MyD88*$^{-/-}$*Trif*$^{-/-}$*Cardif*$^{-/-}$ mice. Dotted lines indicate the LOD for each analyte; values below LOD were assigned half-LOD for visualization. Data from one experiment (*n* = 3–4 mice/group).

(C) Survival, body temperature, and clinical score of WT and *MyD88*$^{-/-}$*Trif*$^{-/-}$*Cardif*$^{-/-}$ mice infected with LCMV (1×10^{6} PFU) and treated 24 h.p.i. with 9E9 (20 µg). Data from one experiment (*n* = 3–4 mice/group).

(D) Serum IFN-α and IFN-β levels in naive WT mice or WT mice infected with a high dose (HD; 1×10^{6} PFU) or low dose (LD; 200 PFU) of LCMV. LOD handling as in (B). Pooled data from two experiments with similar results (*n* = 3–4 mice/group/experiment).

(E) Kaplan–Meier survival curves of WT mice infected with HD or LD LCMV and treated with 9E9 (200 µg) or isotype 24 h.p.i. Pooled from two experiments (n = 4 mice/group/experiment).

(F) Clinical scoring of 9E9-treated mice in (D) assessed using a standardized scoring system (Supplemental Table 1).

(G) Serum levels of IFN-α in naive or LCMV-infected WT mice (1×10^{6} PFU) at days 1, 3, 6, or 9 post infection. Dotted lines indicate the LOD for each analyte. LOD handling as in (B). Results represent data from one experiment (*n* = 5 mice/group).

Data are mean ± SD. Statistical tests: log-rank (A, C, E), two-way ANOVA (A, C, F), Student's t-test (B), and one-way ANOVA (D, G). *p < 0.05, **p < 0.01, ***p < 0.001, ****p < 0.0001.

Supplemental Figure 3. IFN-I regulates FcγRIV expression across myeloid cell subsets

(A) Flow cytometry plots depicting FcγRIV^{+} Ly6C^{high} inflammatory monocytes in blood and spleen of naive and LCMV-infected (1x10^{6} PFU) WT and *Ifnar1*$^{-/-}$ mice 24 h.p.i. Data pooled from 2 independent experiments (*n* = 3 mice/group/experiment).

(B) gMFI and frequencies of FcγRIV expression on inflammatory monocytes (CD11b^{+} CCR2^{+} Ly6C^{high}) gated from lineage-negative cells (Ly6G^{-} CD3^{-} CD19^{-}) in naive and LCMV-infected WT mice (1×10^{6} PFU, 24 h.p.i.). MAR1-5A3 (IFNAR1-blocking antibody, 1 mg) or mouse IgG1 isotype control was administered 5 hours before LCMV infection. Data shown are from two experiments (*n* = 3–5 mice/group/experiment).

(C) gMFI and frequencies of FcγRIV expression on inflammatory monocytes (CD11b^{+} Ly6C^{high}) gated from lineage-negative cells (Ly6G^{-} CD3^{-} CD19^{-}) in LCMV-infected WT and *MyD88*$^{-/-}$*Trif*$^{-/-}$*Cardif*$^{-/-}$ mice (1×10^{6} PFU, 24 h.p.i.). Data shown are from one experiment (*n* = 3–4 mice/group).

(D) gMFI of FcγRIV expression on inflammatory monocytes in naive and LCMV-infected WT mice (1×10^{6} PFU) at days 1, 3, 6, and 9 post infection. Results represent data from one experiment (n = 5 mice/group).

(E) gMFI and frequencies of FcγRIV expression on mast cells (CD117^{+} SSC-A^{high}) gated from lineage-negative cells (Ly6G^{-} CD3^{-} CD19^{-} Ly6C^{-}) in blood of naive or LCMV-infected WT and *Ifnar1*$^{-/-}$ mice (1×10^{6} PFU, 24 h.p.i.). Data are pooled from two independent experiments for infected groups and from one experiment for naive mice (*n* = 3–4 mice/group/experiment).

All data are represented as mean ± SD. Statistical significance was determined using one-way ANOVA (B), student's t-test for (C), or two-way ANOVA (E). *p < 0.05, **p < 0.01, ***p < 0.001, ****p < 0.0001.

Supplemental Figure 4. Neutrophils and mast cells are dispensable for FcγRIV-mediated anaphylaxis

(A) WT and Sash-KO mice infected with LCMV (1×10^{6} PFU) and treated 24 h.p.i. with 9E9 (20 µg). Survival, body temperature and clinical score (Supplemental Table 1) were monitored over time. Data from one experiment (*n* = 3-4 mice/group).

(B) Flow cytometry plots and graphs showing the absolute numbers of inflammatory monocytes and neutrophils in the peripheral blood of mice infected with LCMV-WE (1×10^{6} PFU) at 24 h.p.i., 1A8 (Ly6G-depleting antibody) or isotype control were administered 2 h.p.i. Results show the pooled data from two experiments (*n* = 5 mice/group/experiment).

(C) LCMV-infected mice (1×10^{6} PFU) treated with either the 9E9 antibody (20 µg) or isotype control 24 h.p.i. For some groups, 1A8 (neutrophil-depleting antibody, 200 µg) was administered 2 hours after LCMV infection. Survival, body temperature and clinical score were monitored over time. Results show the pooled data from two independent experiments with similar results (*n* = 4-5 mice/group/experiment).

All data are represented as mean ± SD. Statistical significance was determined using log-rank test for survival (A and C), two-way ANOVA for body temperature and clinical score (A and C), student's t-test (B)., ***p < 0.001.

Supplemental Figure 5. FcγRIV expression on splenic macrophage subsets

(A) Representative immunohistochemistry images of spleen tissue from naive and LCMV-infected WT mice 24 h.p.i. Shown representative images from two independent experiments (*n* = 3 mice/group/experiment). Scale bar = 300 µm.

(B) Graphs represent the gMFI of the FcγRIV expression on red pulp macrophages (RPMs; F4/80^{+} CD11b^{low} of neg. lineage), marginal metallophilic macrophages (MMMs; CD169^{+} TIM4^{+} CD11b^{+} neg. lineage), marginal zone macrophages (MZM; MARCO^{+} TIM4^{+} CD11b^{+} neg. lineage), along with patrolling monocytes in spleen from naive and LCMV-infected WT mice (1x10^{6} PFU) (24 h.p.i.). negative lineage was (Ly6G^{-} CD3^{-} CD19^{-} Ly6C^{-}). Results show the pooled data from two experiments; experiment was repeated three times with similar results. (*n* = 2-3 mice/group/experiment).

(C) Absolute numbers of macrophages, inflammatory monocytes and neutrophils in spleen of LCMV-infected WT mice. Clodronate or control liposomes (200 µL) were administered 2 hours prior to LCMV infection. Results show the pooled data from two independent experiments with similar results (*n* = 3-5 mice/group/experiment).

(D) WT mice infected with LCMV (1×10^6 PFU) were treated 24 h.p.i. with 9E9 (20 µg) or isotype control. Some mice received Clodronate (200 µL) 2 h prior to infection to deplete phagocytes. Survival, body temperature and clinical score (Supplemental Table 1) were monitored over time. Data pooled from two experiments (*n* = 3–5 mice/group/experiment).

All data are represented as mean ± SD. Statistical significance was determined using student's t-test (B and C), or log-rank test for survival (D), two-way ANOVA for body temperature and clinical score (D), *p < 0.05, **p < 0.01, ****p < 0.0001

Supplemental Figure 6. Red pulp macrophages are dispensable for FcγRIV-dependent anaphylaxis

(A) Absolute numbers of RPMs ($F4/80^+$ $CD11b^{low}$) gated from negative lineage ($Ly6G^-$ $CD3^-$ $CD19^-$) in spleen of naive *Vav1*-Cre^{WT} x *Spic*$^{fl/fl}$ and *Vav1*-Cre^{Tg} x *Spic*$^{fl/fl}$. (*n* = 4 mice/group).

(B) Representative immunohistochemistry images of spleen tissue from naive and LCMV-infected (1×10^6 PFU) *Vav1*-Cre^{WT} x *Spic*$^{fl/fl}$ and *Vav1*-Cre^{Tg} x *Spic*$^{fl/fl}$ mice 24 h.p.i. Data represents two independent experiments (*n* = 2-4 mice/group/experiment). Scale bar = 100µm.

(C) LCMV-infected (1×10^6 PFU) *Vav1*-Cre^{WT} x *Spic*$^{fl/fl}$ and *Vav1*-Cre^{Tg} x *Spic*$^{fl/fl}$ mice were treated with 9E9 (20 µg) or isotype control 24 h.p.i. Survival, body temperature and clinical score were monitored over time. Pooled from two experiments (*n* = 3–5 mice/group/experiment).

All data are represented as mean ± SD. Statistical significance was determined using log-rank test for survival, two-way ANOVA for body temperature and clinical score, student's t-test (A). ****p < 0.0001

Supplemental Figure 7. Inflammatory monocytes are required for FcγRIV-driven anaphylaxis

(A–B) Flow cytometry plots and graphs showing frequencies of inflammatory monocytes and neutrophils in peripheral blood of mice infected with LCMV-WE (1×10^6 PFU) at 24 h.p.i. Antibody treatments were administered at 2 h.p.i.: (A) RB6-8C5 (anti-GR1) or (B) MC21 (anti-CCR2), alongside respective isotype controls. Graphs depict absolute numbers of the indicated cell populations. Data are pooled from two independent experiments (*n* = 4–5 mice/group/experiment).

(C) WT and *CCR2*$^{-/-}$ mice were infected with LCMV (1×10^6 PFU) and treated 24 h.p.i. with the 9E9 (20 µg). Survival, body temperature and clinical score (Supplemental Table 1) were monitored in all treated groups. Results show data from two experiments (*n* = 4 mice/group/experiment).

(D) Flow cytometry plots showing $FcγRIV^+$ and $FcγRIV^-$ inflammatory monocytes in peripheral blood and bone marrow of naive and LCMV–infected mice (1×10^6 PFU, 24 h.p.i.). Graphs depict absolute numbers of $FcγRIV^+$ and $FcγRIV^-$ inflammatory monocyte subsets in WT, *CCR2*$^{-/-}$, or MC21-treated WT mice. MC21 (anti-CCR2, 35 µg) was administered 2 h.p.i. Shown data are from one experiment (*n* = 5 mice/group), representative of two independent experiments with similar results.

All data are represented as mean ± SD. Statistical significance was determined using log-rank test for survival (C), two-way ANOVA for body temperature and clinical score (C), student's t-test (A and B), one-way ANOVA for (D). *p < 0.05, **p < 0.01, ***p < 0.001, ****p < 0.0001.

Supplemental Figure 8. Different viruses induce FcγRIV upregulation on inflammatory monocytes

(A-B) Flow cytometry plots (A) show the percentages of FcγRIV$^+$ inflammatory monocytes (CD11b$^+$ Ly6C^{high}) gated from negative lineage (Ly6G$^-$ CD3$^-$ CD19$^-$) in peripheral blood and spleen of K18-hACE2 mice 48 hours after intranasal infection with SARS-CoV-2 (10^4 PFU). Graphs (B) depict the gMFI of FcγRIV expression on inflammatory monocytes in blood and spleen from naive and SARS-CoV-2–infected mice. Data are from one experiment (*n* = 3–4 mice/group).

(C) Graphs showing the percentage and gMFI of FcγRIV on inflammatory monocytes in peripheral blood of naive and infected WT mice 24 hours after infection with herpes simplex virus 1 (HSV-1; 1×10^6 PFU) or influenza A virus (1×10^6 PFU). Data are from one experiment (*n* = 4–5 mice/group).

All data are represented as mean ± SD. Statistical significance was determined using Student's t-test (A and B) or one-way ANOVA (C). *p < 0.05, **p < 0.01, ***p < 0.001, ****p < 0.0001

Supplemental Figure 9. Mast cells and FcγRIII are dispensable for PAF production upon FcγRIV activation

(A) Serum PAF levels in LCMV-infected *Fcgr3*$^{+/+}$ and *Fcgr3*$^{-/-}$ mice (1×10^6 PFU) 20 min after intraperitoneal 9E9 (200 µg) or isotype treatment 24 h.p.i. Data representative of two experiments (*n* = 4 mice/group/experiment).

(B) LCMV-infected WT mice treated with 9E9 (200 µg) or isotype 24 h.p.i., with or without Clodronate pre-treatment. Serum PAF measured 20 min after intraperitoneal 9E9 (200 µg) or isotype administration. Data pooled from two experiments (*n* = 2–3 mice/group/experiment).

(C) Serum PAF levels in LCMV-infected WT and Sash-KO mice (1×10^6 PFU) 24 h.p.i. Serum was collected 20 minutes after 9E9 or isotype antibody treatment (20 µg). Data from one experiment (*n* = 3–4 mice/group).

All data presented as mean ± SD. Statistical significance: one-way ANOVA. *p < 0.05, **p < 0.01, ***p < 0.001, ****p < 0.0001.

Supplemental Figure 10. Inflammatory monocytes are contributing to FcγRIV-dependent systematic active anaphylaxis after infection

(A) Survival, body temperature, and clinical score in LCMV-infected *Fcgr3*$^{-/-}$ mice treated with 35 µg αCCR2 (MC-21) to deplete inflammatory monocytes or isotype control 2 h.p.i. ASA was induced by intravenous injection of 200 µg BSA upon immunization schedule mentioned in (Figure 10A). Results show data from one experiment (*n* = 6 mice/group).

All data presented as mean ± SD. Statistical significance: two-way ANOVA or log-rank test. *p < 0.05, **p < 0.01.
